# Computational Appraisal of Silver Nanocluster Evolution on Epitaxial Graphene: Implications for CO Sensing

**DOI:** 10.1021/acsomega.1c03577

**Published:** 2021-09-15

**Authors:** Ivan Shtepliuk, Rositsa Yakimova

**Affiliations:** Department of Physics, Chemistry and Biology-IFM, Linköpings Universitet, 58183 Linköping, Sweden

## Abstract

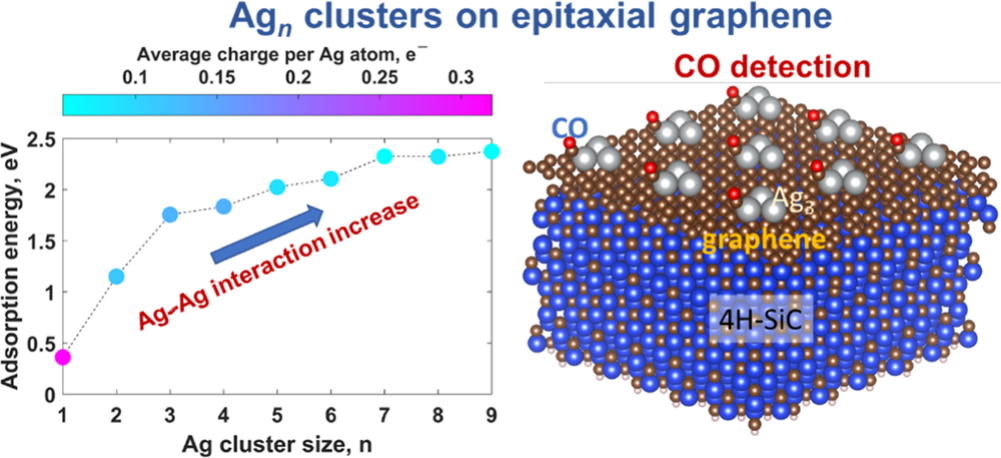

Early stages of silver nucleation on a two-dimensional (2D) substrate, here, monolayer epitaxial graphene (MEG) on SiC, play a critical role in the formation of application-specific Ag nanostructures. Therefore, it is of both fundamental and practical importance to investigate the growth steps when Ag adatoms start to form a new phase. In this work, we exploit density functional theory to study the kinetics of early-stage nuclei Ag_*n*_ (*n* = 1–9) assembly of Ag nanoparticles on MEG. We find that the Ag_1_ monomer tends to occupy hollow site positions of MEG and interacts with the surface mainly through weak dispersion forces. The pseudoepitaxial growth regime is revealed to dominate the formation of the planar silver clusters. The adsorption and nucleation energies of Ag_*n*_ clusters exhibit evident odd–even oscillations with cluster size, pointing out the preferable adsorption and nucleation of odd-numbered clusters on MEG. The character of the interaction between a chemisorbed Ag_3_ cluster and MEG makes it possible to consider this trimer as the most stable nucleus for the subsequent growth of Ag nanoparticles. We reveal the general correlation between Ag/MEG interaction and Ag–Ag interaction: with increasing cluster size, the interaction between Ag adatoms increases, while the Ag/MEG interaction decreases. The general trend is also supported by the results of charge population analysis, according to which the average charge per Ag adatom in a Ag_*n*_ cluster demonstrates a drastic decrement with cluster size increase. 2D–3D structural transition in Ag_*n*_ clusters was investigated. We anticipate that the present investigation is beneficial by providing a better understanding of the early-stage nucleation of Ag nanoparticles on MEG at the atomic scale. Specific interaction between odd-numbered Ag clusters preadsorbed onto the MEG surface and carbon monoxide (CO) as well as clusters’ stability at 300 K is discussed in terms of sensing applications.

## 1 Introduction

Silver nanostructures are extensively appealing due to their catalytic,^1,2^ magnetic,^3,4^ antibacterial,^5,6^ and plasmonic properties.^7,8^ These properties are very sensitive to Ag nanostructures’ morphology that is strongly determined by the growth interface,^9^ the nominal metal–substrate interaction,^10^ and the temperature,^11^ respectively. By controlling the chemical nature, size, and shape of supported Ag nanoislands, it is possible to tune the plasmon resonance frequency,^12,13^ catalytic performance,^14,15^ and magnetic moment.^16,17^ In this regard, a profound knowledge of Ag nucleation and early stages of Ag nanostructure growth is of fundamental importance for the achievement of desired electronic, magnetic, optical, and chemical properties.

Apart from the intrinsic properties of Ag nanoparticles, from the technological point of view, it is imperative to ensure the reproducible and uniform growth of Ag nanostructures on large-area substrates, which is a key prerequisite for boosting their utility for real applications. A novel tendency is the exploration of atomically flat substrates, such as monolayer epitaxial graphene (MEG) on SiC.^18^ Indeed, in contrast to roughened substrates, MEG can act as a defectless support for the highly controlled and ordered arrangement of Ag nanoislands, thereby preventing possible spatial inhomogeneity effects induced by the undesired interaction between Ag species and structural imperfections. Concomitantly, it is challenging to perform growth on dangling bond-free surfaces such as MEG in order to gain new properties and benefit from them in both fundamental and applicational aspects. Therefore, the interplay between silver and epitaxial graphene, as a two-dimensional (2D) substrate, is an intriguing subject in applied physics.^19−30^

According to state of the art, there are two major approaches to capitalize the benefits of the combined Ag/MEG/SiC system. One is the direct deposition of silver on the MEG surface, followed by the subsequent nucleation of Ag nanoparticles.^19−26^ MEG-supported Ag nanoparticles have been used for (i) catalytic nanoparticle-assisted oxygen etching of epitaxial graphene/SiC(0001) to form oxygen-terminated zigzag ribbons with 1D metallic edges,^19^ (ii) improving the sensitivity of MEG/6H-SiC toward H_2_ gas detection,^20^ (iii) inducing surface-enhanced Raman scattering phenomena at the MEG surface,^21,22^ (iv) n-type doping of MEG,^23−25^ and (v) tuning the magnetic properties of MEG via Ag substitutional doping.^26^ The second approach is Ag intercalation with the zero-graphene layer (buffer layer),^27−30^ which has two major consequences: (i) formation of n-doped quasi-freestanding monolayer graphene (QFMG) through buffer layer decoupling from the SiC surface and (ii) encapsulation of Ag nanoparticles within the QFMG–SiC confined space which prevents undesirable agglomeration and degradation phenomena. The goal of more recent investigations was to achieve self-assembling of large-area continuous 2D silver films beneath QFMG, which are believed to have semiconducting properties in contrast to bulk silver. Synthesis of a 2D Ag monolayer forming through the rearrangement of intercalated Ag species has been experimentally demonstrated by Briggs et al.^29^ and Rosenzweig and Starke,^30^ respectively. Apparently, a reliable control of Ag morphology within both considered approaches requires a holistic theoretical understanding of Ag/MEG interaction’s influence on the initial atomistic-level growth of Ag nanostructures. Computational study could not only help to complement and interpret experimental results but also to design Ag/MEG/SiC nanoarchitectures with the desired performance. However, despite the ever-increasing interest in this material system, nucleation and growth of silver on MEG are still scarcely investigated from a fundamental point of view. In the current paper, to untangle the mechanisms underlying the early stages of the Ag growth on MEG, we are aiming to explore the adsorption, surface diffusion, and nucleation phenomena related to the interfacing of small planar silver clusters (from Ag_1_ to Ag_9_) to MEG/SiC. These clusters can be regarded as the initial nanoscale building blocks (early-stage nuclei) for the growth of the large-area 2D silver layer. Therefore, in this paper, we mainly focus on the atomistic processes behind 2D Ag cluster formation. However, the formation of small-sized three-dimensional (3D) clusters as a fundamental preprocess underlying the growth of 3D metal nanoparticles and nanoislands (via Ostwald ripening)^31,32^ will also be discussed. This paper emphasizes the benefits of the Ag_*n*_/MEG/SiC hybrid materials for CO sensing.

## 2 Results and Discussion

### 2.1 Diffusion of a Single Ag Adatom (Ag_1_) on MEG

**Figure 1:**
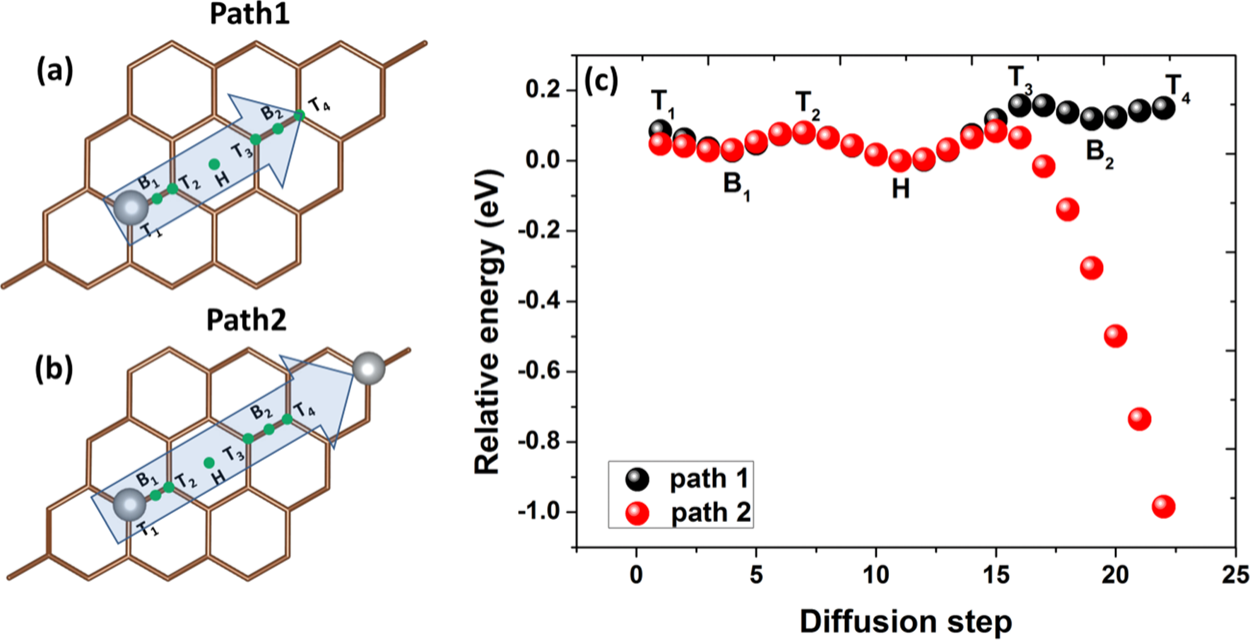
Sketch of the path for the diffusion of a single Ag adatom on MEG (a) in the absence and (b) in the presence of a preadsorbed Ag adatom. Note: T_1,2_ (B_1_) sites are not equivalent to T_3,4_ (B_2_), as in the case of freestanding graphene. This is due to the presence of the buffer layer beneath graphene. (c) Potential-energy profiles corresponding to the migration of Ag along both considered diffusion paths over MEG. Relative energy (RE) of the systems was calculated as follows: , where *E*_tot_^Ag/MEG^ is the total energy of the system at each diffusion step, while  is the total energy of the system when Ag occupies the hollow site. In fact, the total energy was normalized to , so that the most stable adsorption site has an energy of 0 eV and less stable sites have positive energies. Meanwhile, the negative energy suggests a strong attractive interaction between the newly coming Ag atom and preadsorbed Ag adatom.

Generally, there are three inequivalent high-symmetry adsorption sites on the graphene surface:^33^ top site T (above the carbon atom), bridge site B (above the center of the carbon–carbon bond), and hollow site H (above the center of the hexagonal ring). Therefore, to get a full picture of the diffusion process, it is necessary to construct the diffusion path passing via all possible adsorption positions. In the case of epitaxial graphene, the presence of the buffer layer changes the surface energetics. Since the buffer layer is corrugated and the buffer layer-first graphene layer pair resembles, to some extent, AB-stacked bilayer graphene, one can expect the appearance of more than three unique adsorption sites^22^ and, as a result, more asymmetrical potential energy profiles. Figure 1a represents the chosen diffusion path for the migration of a single Ag atom immediately after its adsorption on the MEG surface (see also Figure S1, Supporting Information). As demonstrated in Figure 1c, the potential energy profile includes two local minima associated with Ag migration above the bridge sites B_1_ and B_2_ and global minimum at the H position. Whenever the as-deposited Ag species reach the MEG surface, they will preferentially diffuse toward the hollow site position. Ag atoms will avoid occupation of the unfavorable on-top sites T_1_–T_4_. Furthermore, there is an energy barrier of 79 meV (157 meV) that must be overcome to escape from the hollow site position toward B_1_ (B_2_) sites, while the energy difference between B sites and T sites ranges from 30 to 55 meV.

**Figure 2:**
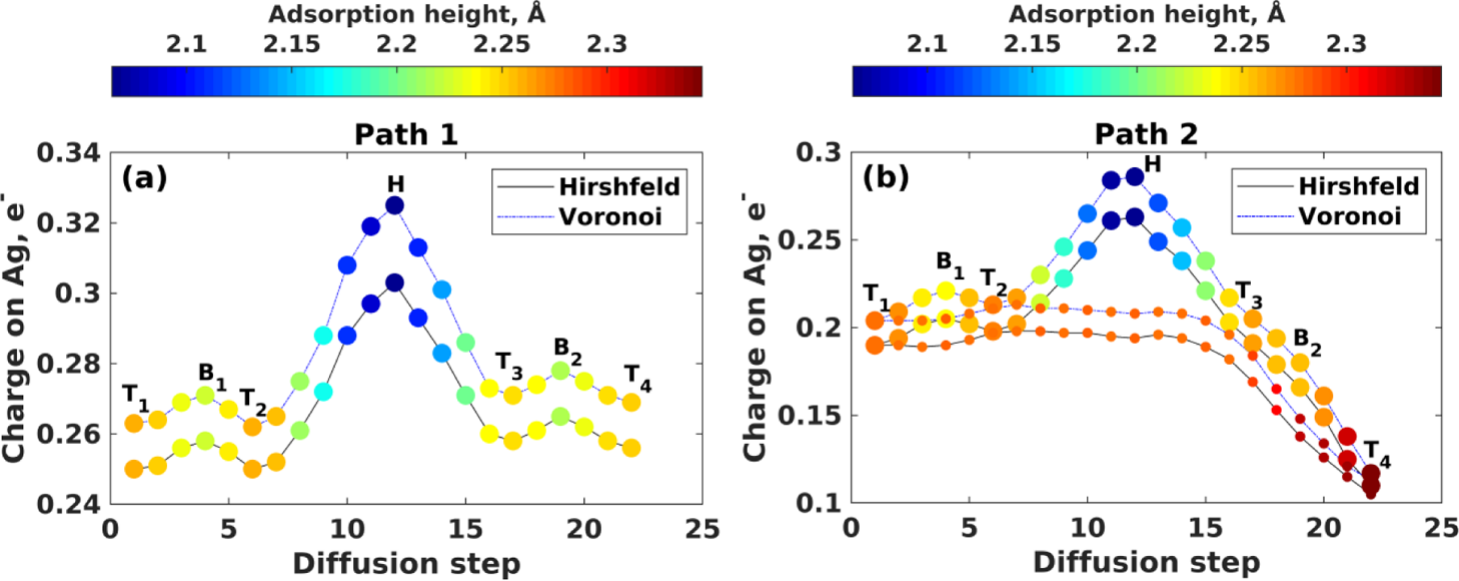
Fluctuations of the effective charge on the Ag adatom at each stage of the diffusion path for Ag movement on MEG (a) in the absence and (b) in the presence of a preadsorbed Ag adatom. Smaller balls on (b) represent the effective charge on the preadsorbed Ag adatom. The charge population analysis was performed by using Hirshfeld and Voronoi schemes, respectively. Positive sign of charge on the Ag adatom means that it donates extra electrons to the MEG.

The position-dependent charge on an Ag adatom color-coded according to the adsorption height (Figure 2a) confirms that being captured by a hollow site, the Ag adatom donates the largest number of electrons to graphene (Hirshfeld and Voronoi charges are 0.303 and 0.325 e^–^, respectively) and is characterized by the shortest adsorption height (2.06 Å). Based on the present results, it is reasonable to assume that after the deposition of the Ag adatom, it will be trapped at the hollow site position and may serve as a nucleation site for small Ag nanoparticles. It is worth noting that the presence of the preadsorbed silver atom at the MEG surface (Figure 1b) substantially affects the diffusion path due to the attractive interatomic interaction between Ag species. The energy barrier which is necessary for escaping from the trapping center (hollow site) in the presence of another Ag adatom was found to be comparable to the same for the diffusion of a single Ag adatom (∼80 meV). After reaching the critical Ag–Ag distance (5.19 Å), the potential of the interaction between two Ag adatoms becomes significantly attractive, so the resulting forces push out Ag atoms from the surface and eventually decrease the charge transfer to graphene. This is evidenced by the increased Ag adsorption height and reduced remaining charge on the Ag adatom (Figure 2b) after reaching the critical distance. From the practical point of view, this means that the preadsorbed Ag adatoms will create gradients in the surface energy, thereby providing an additional driving force for the surface movement of Ag atoms to form a nucleus. The motion of a single Ag atom toward another preadsorbed single Ag atom on the MEG surface will result in the formation of the Ag_2_ dimer parallel to the MEG surface, which can be classified as small-sized early-stage 2D nuclei. After the formation of the nuclei on the MEG surface, they can further capture nearby surrounding Ag adatoms to form larger nanoclusters and nanoislands.

### 2.2 Adsorption and Nucleation of Ag_*n*_ Clusters on MEG

**Figure 3:**
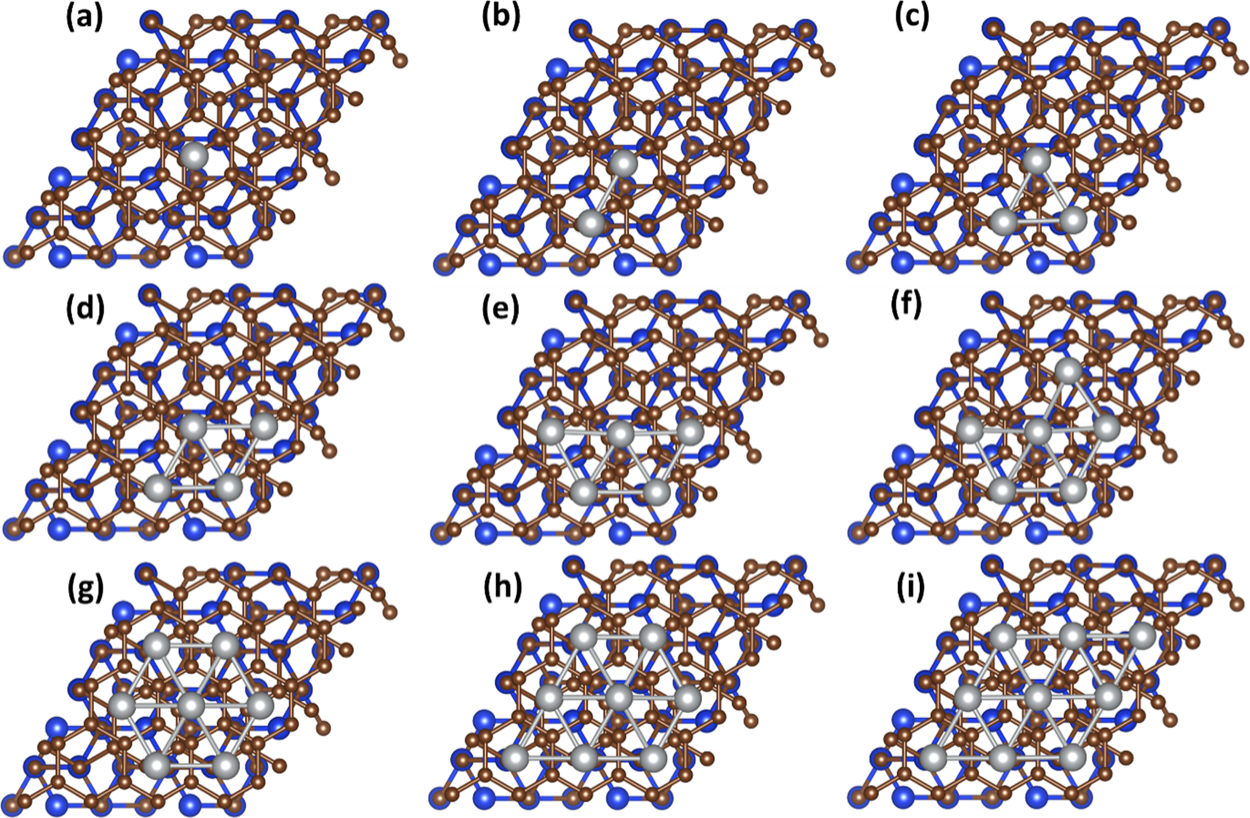
(Top view) Optimized geometrical structures of 2D Ag_*n*_ clusters supported by MEG: from Au_1_ (a) to Au_9_ (i), respectively. Such geometrical configuration is assigned to the case of the pseudoepitaxy. Blue, brown, and grayish balls designate Si, C, and Ag atoms, respectively.

Although the above-mentioned results give some hints on the nature of Ag growth on MEG, the overall picture still needed to be further clarified. An in-detail investigation of Ag-related adsorption and nucleation processes on the MEG surface will provide critical knowledge on early stages of the Ag nanoparticle growth process. Starting from the adsorption of the Ag_1_ monomer, we next extend our studies to Ag_*n*_ (*n* = 2–9) clusters. Two different cases are examined:

(i) for all Ag adatoms initially located above H sites, only vertical displacements in the *z* direction are allowed. Thus, spontaneous in-plane (*x*, *y*) atomic movements are forbidden. This case can be, to some extent, referred to a partial *pseudomorphy*. Generally, pseudomorphy takes place only where the deposited atoms occupy all available template positions,^9,34^ forming completely coherent bonds. The main prerequisite for that is the prevailing of the deposit–template interaction energy over the deposit–deposit lateral interaction energy. For the sake of completeness, we performed additional calculations and investigated the pseudomorphy of Ag at bridge (B) and top (T) sites.
(ii) all Ag adatoms positioned at hollow sites are enabled to relax in all three directions. Such geometrical configuration can be, formally, ascribed to *pseudoepitaxy*.^9,34^ In this case, the deposit–deposit lateral interaction strength is assumed to be larger than the deposit–template interaction strength and thus Ag atoms can more easily migrate laterally to form the Ag overlayer with Ag–Ag bonds that are very close to those for bulk Ag. In other words, we expect the formation of Ag clusters on MEG. The upper limit of the Ag_*n*_ cluster size was chosen to be 9 to cover all nine hexagonal rings of the 4 × 4 supercell with Ag adatoms. In fact, this value is in reasonable agreement with the geometrical criterion, which describes how many Ag adatoms can be accommodated at the MEG surface. Indeed, the interrelationship between the number of C atoms of graphene top layer (*n*_C_) and the maximum acceptable amount of Ag adatoms that could be accommodated at this surface (*n*_Ag_ = *n*_C_ + δ*n*) can be expressed by using the following equation^9^

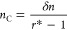
1where r* is the ratio of C to Ag atomic radii, equal to 0.4375. Bearing in mind that the 4 × 4 supercell of MEG contains 32 carbon atoms, the solution of eq 7 gives δ*n* = −18 and *n*_Ag_ = 14. Thus, the maximal cluster size in our work does not exceed *n*_Ag_. Finally, the optimized geometries of MEG after Ag_*n*_ deposition are summarized for pseudomorphy in Figures S2–S7 (Supporting Information) and for pseudoepitaxy in Figures 3 and S8 (Supporting Information). According to our estimations, pseudoepitaxial structures are more stable compared to all pseudomorphic ones (Figure S9, Supporting Information), which is confirmed by their lowest total energies within the whole *n* range from 1 to 9.

**Figure 4:**
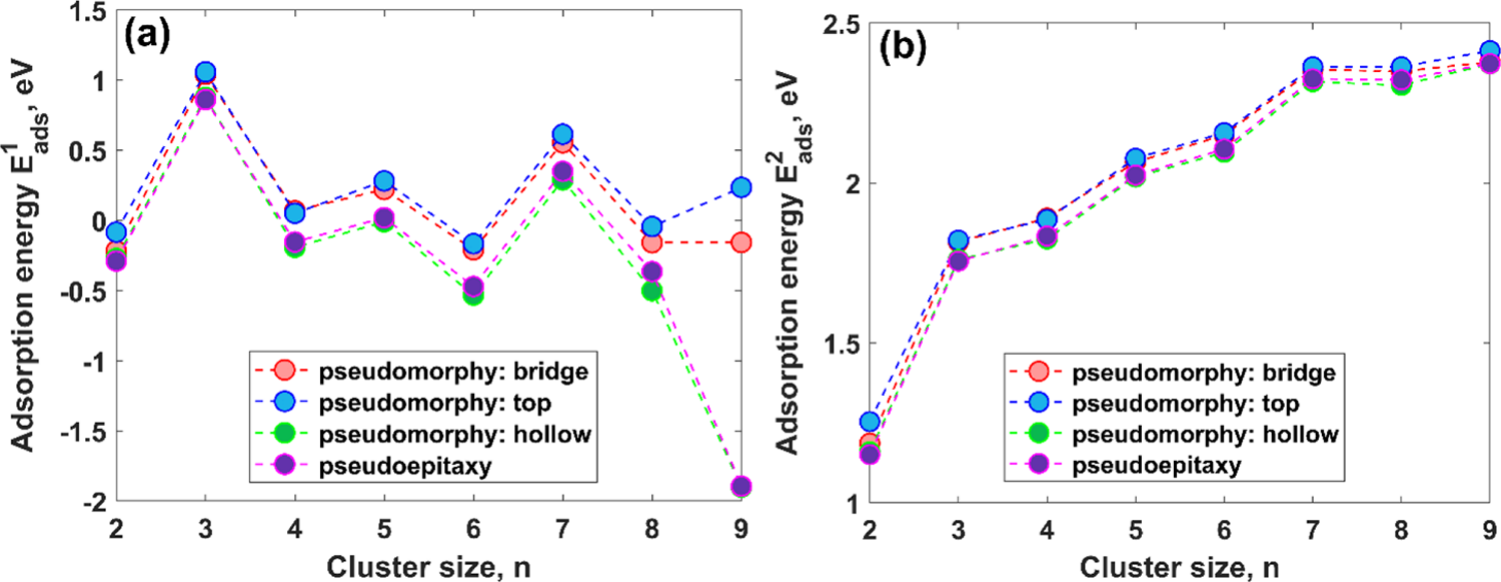
Dependences of the calculated adsorption energies of Ag_*n*_ clusters on the cluster size *n*: (a) *E*_ads_^1^ vs *n* and (b) *E*_ads_^2^ vs *n*, respectively.

It is obvious that the structure of the pseudomorphic Ag/MEG system is remarkably similar to that observed for the pseudoepitaxial Ag/MEG (except the adsorption height), even though for the pseudomorphy case, any lateral displacement for silver adatoms was forbidden. That is confirmed by the same trends for the adsorption energies (*E*_ads_^1^ and *E*_ads_^2^), as demonstrated in Figure 4a,b, respectively. The dependence of *E*_ads_^1^ and *E*_ads_^2^ adsorption energies on cluster size for pseudomorphy closely resembles the same dependences for pseudoepitaxy, the only distinction being that the interaction between MEG and pseudomorphical Ag clusters formed at bridge and top sites is weaker than that between MEG and pseudomorphical and pseudoepitaxial Ag clusters formed at hollow sites. The similarity between pseudomorphy at hollow sites and pseudoepitaxy can be comprehended by the similarity in adsorption positions. In both considered cases, the Ag atoms occupy the hollow sites of the graphene lattice, with the difference that in the pseudoepitaxy case, all Ag atoms may move in all directions, while in the pseudomorphy case, the motion of Ag atoms is only allowed in the *z*-direction (normal to the MEG surface) during the relaxation process.

**Figure 5:**
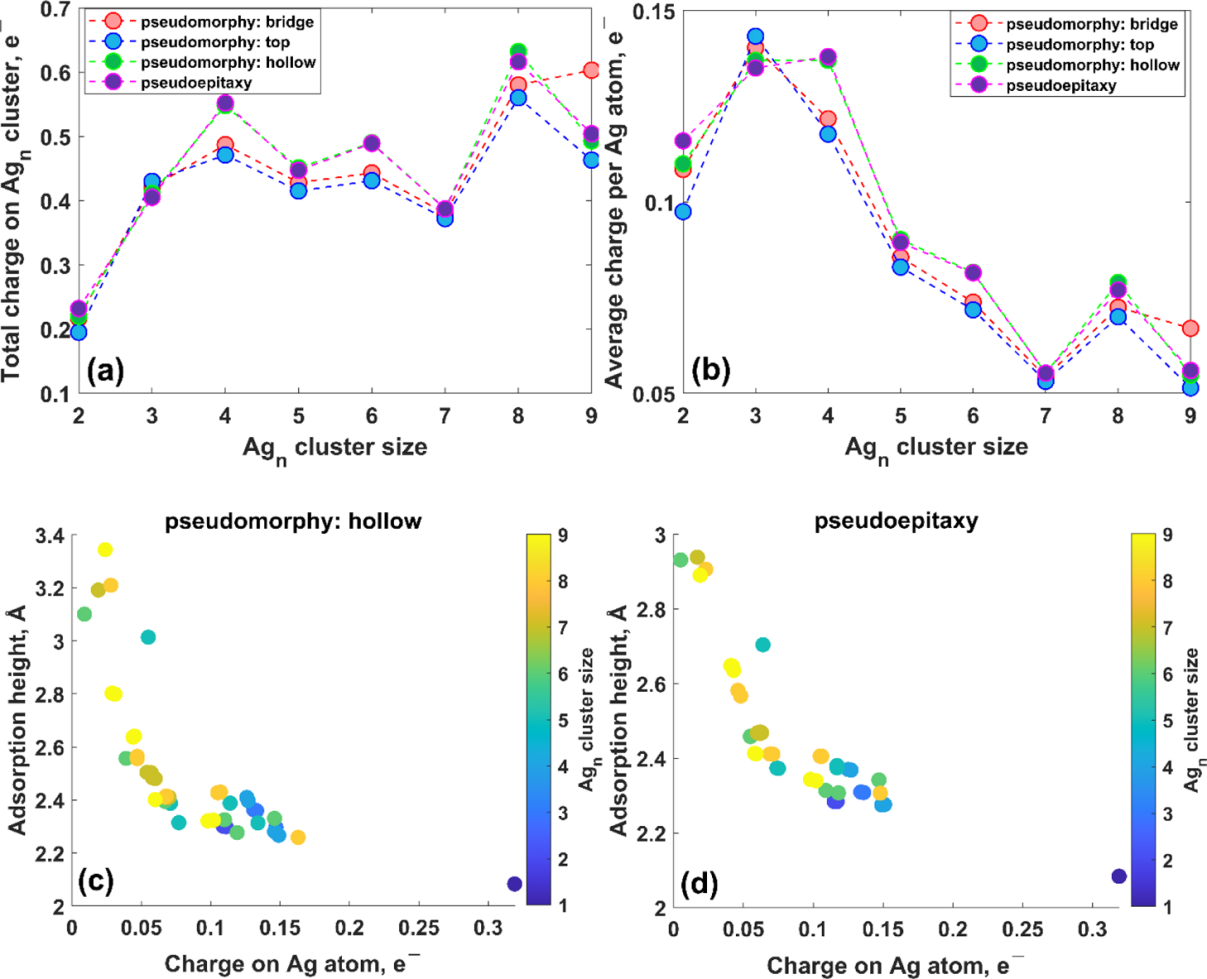
(a) Dependence of the effective (Voronoi) charge transfer on the Ag_*n*_ cluster on cluster size for two considered cases: pseudomorphy and pseudoepitaxy. (b) Average charge per Ag atom vs the cluster size. Correlation between the adsorption height of the Ag adatom in the Ag_*n*_ cluster and effective charge on the same atom: (c) pseudomorphy and (d) pseudoepitaxy, respectively.

The adsorption energy *E*_ads_^1^, which is the main indicator of Ag_*n*_/MEG interaction strength, also demonstrates strong odd–even oscillations with local maxima at *n* = 1, 3, 5, and 7. It should be noted that the energies corresponding to these local maxima are positive, pointing out that the adsorption process is thermodynamically favorable. In contrast to odd-numbered Ag_*n*_ clusters, the adsorption of even-numbered Ag_*n*_ clusters with *n* = 2, 4, and 6 is an energetically unfavorable process that is evidenced by the gradual increase of negative values of *E*_ads_^1^. However, in the meantime, *E*_ads_^1^ of the largest odd-numbered Ag_9_ cluster becomes negative, probably due to the large energy penalty that needs to be paid to accommodate a large number of Ag adatoms on the MEG surface. Such odd–even oscillations of the adsorption energy can be explained by the fact that odd-numbered clusters having unpaired electrons can be more easily ionized, providing stronger bonding to the substrate. Based on the results of Voronoi population analysis, we revealed that Ag_*n*_ clusters electronically dope MEG (total cluster charge varies from 0.23 to 0.61 e^–^), but no obvious correlation between *E*_ads_^1^ and the total cluster charge was found (Figure 5a). On the other hand, the magnitude of the average charge per Ag atom in a Ag_*n*_ cluster shows more pronounced dependence on the cluster size (Figure 5b), which is consistent with cluster size-dependent *E*_ads_^2^ (Figure 4b). Indeed, as was mentioned before, the adsorption energy *E*_ads_^2^ includes two components: cohesive energy of the Ag cluster and the strength of the interaction between MEG and the Ag clusters.

According to our estimations, *E*_ads_^2^ is positive within a broad range of cluster sizes and demonstrates gradual increment with cluster size increase with tendency to saturation. Such a behavior can be explained by the fact that the adsorption energy components demonstrate opposing trends. On one hand, the deposit–substrate interaction oscillates with the cluster size so that the adsorption of large clusters becomes energetically unfavorable (see Figure 4a). On the other hand, the cohesive energy of Ag_*n*_ clusters becomes more negative with cluster size, suggesting an increase in the cluster stability (as demonstrated in Table 1). In other words, weakening the metal–support interaction makes Ag–Ag distances and cohesive energy comparable to those of bulk Ag. As a result, the average charge on the Ag atom in the Ag_*n*_ cluster decreases (Figures 5c,d and S10, Supporting Information), and finally, a repulsive force pushes out Ag adatoms from the MEG surface, which is confirmed by the adsorption height increase.

**Table 1:** Calculated Parameters for Ag_*n*_ (*n* = 1–9) Cluster Adsorption and Nucleation on MEG

*n*	adsorption energy, eV	deformation energy of Ag_*n*_, eV	deformation energy of MEG, eV	cohesive energy, eV	nucleation energy, eV	*E*_ads_^1^/*E*_coh_ ratio
1	0.361	0.0007	0.040	a–2.95		–0.122
2	–0.290	0.056	0.043	–1.296	–1.578	0.224
3	0.860	0.037	0.081	–1.468	–2.603	–0.586
4	–0.153	0.112	0.104	–1.872	–1.710	0.082
5	0.019	0.113	0.096	–2.020	–2.423	–0.010
6	–0.471	0.173	0.132	–2.184	–2.151	0.215
7	0.350	0.088	0.107	–2.275	–3.280	–0.153
8	–0.364	0.148	0.118	–2.368	–1.941	0.153
9	–1.895	1.895	0.098	–2.583	–2.417	0.733

a Cohesive energy of bulk silver.^35,36^

By comparing Figure 5c,d (see also Figure S10, Supporting Information), it is clearly seen that the adsorption heights for Ag adatoms belonging to pseudomorphic Ag_*n*_/MEG systems are higher compared to those belonging to the pseudoepitaxial Ag_*n*_/MEG system, thereby suggesting that the pseudomorphic growth regime causes the formation of more corrugated silver overlayers on MEG. Taking this into account, we will next focus only on the pseudoepitaxy case.

**Figure 6:**
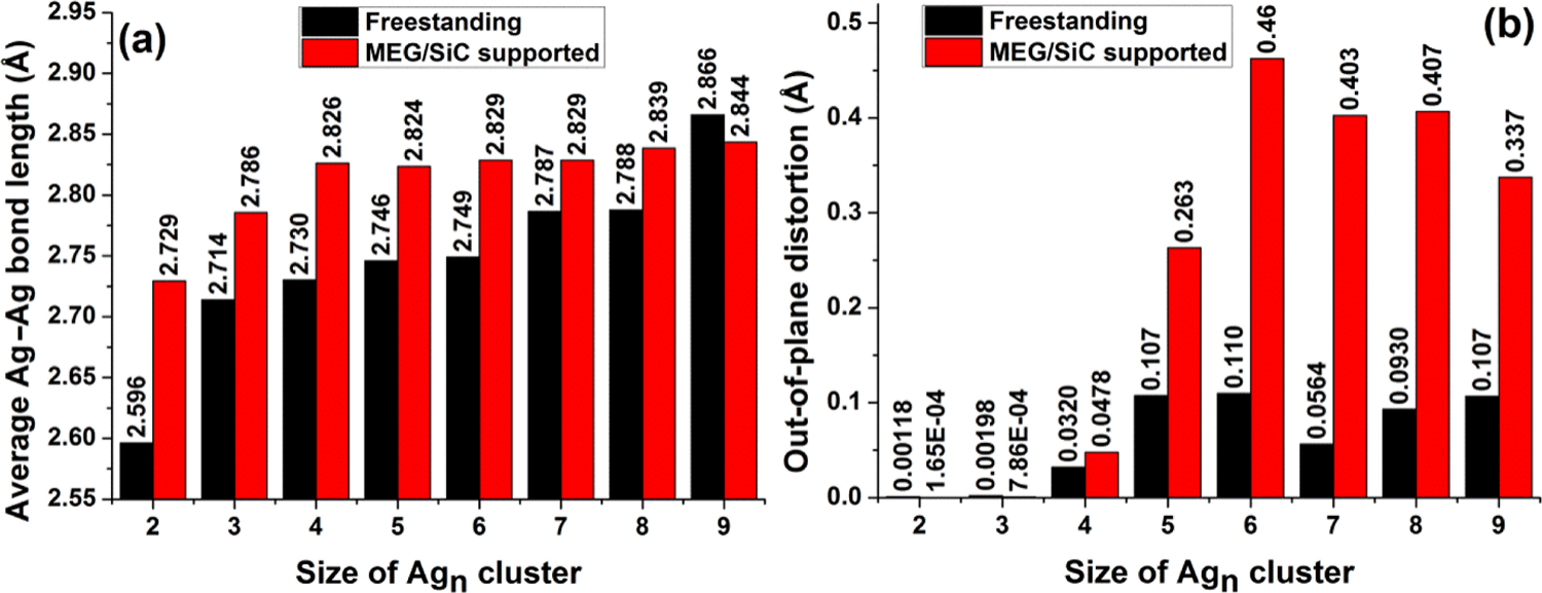
Comparative bar charts of (a) average Ag–Ag bond length and (b) out-of-plane distortion in freestanding and MEG-supported Ag_*n*_ clusters, respectively.

To study further the effect of the MEG substrate on the structure of Ag_*n*_ clusters, we analyzed and compared the average Ag–Ag bond lengths for neighboring atoms within the freestanding (note: the optimized structures of freestanding Ag_*n*_ clusters are shown in Figure S11, Supporting Information) and supported Ag_*n*_ clusters (Figure 6a) and out-of-plane distortions for the corresponding clusters (Figure 6b).

As shown in Figure 6a, due to the metal–substrate interaction and the existence of restricted adsorption sites, the average Ag–Ag bond length in supported Ag_*n*_ clusters is, in almost all cases, larger than that in freestanding clusters. The difference is strongly decreased for large clusters, suggesting that the average Ag–Ag distance tends to the value of a freestanding Ag monolayer. In addition, we observed a substantial cluster-size-dependent out-of-plane distortion of supported clusters with respect to freestanding counterparts (Figure 6b). It is obvious that the stabilization of Ag_*n*_ clusters on the MEG surface is achieved through a deformation process. The estimated values of the deformation energies are listed in Table 1. Concomitantly, the energy penalty that needs to be paid to deform MEG for the stabilization of Ag clusters on the surface is much less.

By estimating the nucleation energy, we revealed that the nucleation process on MEG is energetically favorable for all considered clusters Ag_*n*_ (*n* = 2–9), indicating that the critical cluster size for Ag nanoparticle nucleation on MEG is equal to 2. Another important finding is that the nucleation energy has prominent size-dependent odd–even oscillations (Table 1). Particularly, the nucleation energy of each subsequent odd-numbered Ag_*n*_ clusters (*n* = 3, 5, 7, and 9) is larger than that of preceding even-numbered Ag_*n*_ clusters with *n* = 2, 4, 6, and 8, respectively. From the experimental point of view, such a difference means that the small odd-numbered Ag clusters rather than even-numbered counterparts are more likely to act as nucleation centers for further Ag nanoparticle growth. Being preadsorbed on the MEG surface, these clusters can more easily capture newly incoming Ag atoms to form larger clusters or islands. As has been mentioned, only Ag_*n*_ clusters with *n* = 1, 3, 5, and 7 have a positive adsorption energy, *E*_ads_^1^. Interestingly, the values of the adsorption energy for Ag_*n*_ clusters with *n* = 1, 5, and 7 are below the upper limit of physisorption (0.5 eV), indicating that their interaction with MEG is mainly governed by weak van der Waals forces. Meanwhile, the adsorption of the Ag_3_ cluster is comparatively strong (adsorption energy of 0.860 eV), which can be ascribed to the chemisorption case. Taking all obtained results into account, one can conclude that the Ag_3_ clusters have the largest probability to stick to MEG, thereby causing the growth of high-density Ag islands. The formation of silver nanoclusters by the assembly of several sputtered Ag atoms before they reach the substrate surface has been previously shown by Mondal and Bhattacharyya.^37^ This confirms our assumption that already-formed Ag_3_ clusters can be directly deposited on the MEG surface, acting as the smallest building blocks during the early stages of Ag nanoparticle growth.

To acquire additional evidence of island-like growth of Ag films on MEG, we estimated the *E*_ads_^1^/*E*_coh_ ratio (see the Table 1), which is believed to be a reliable descriptor of the growth mode for different metals on graphene.^10^ Disregarding the unfavorable adsorption cases, the values of |*E*_ads_^1^/*E*_coh_| ratio for Ag_1_, Ag_3_, Ag_5_, and Ag_7_ clusters are estimated to be 0.122, 0.586, 0.01, and 0.153 eV, respectively. The predicted ratios are quite low, which implies that the Ag growth mode may be similar to the Volmer–Weber growth mechanism,^10^ supporting the formation of small islands at the initial growth stages. Furthermore, the restricted Ag adsorption on top site (T) positions and preferred hollow site-to-hollow site migration of Ag on MEG hinders, to some extent, the Ag adatom diffusion on MEG, thereby causing the formation of a large amount of nucleation centers. The deposition of preformed Ag_3_ clusters that strongly interact with the MEG surface can provide more beneficial conditions for the subsequent nucleation of small nanoislands compared to the deposition of single Ag adatoms.

### 2.3 Formation of 3D Silver Clusters of the MEG Substrate

**Figure 7:**
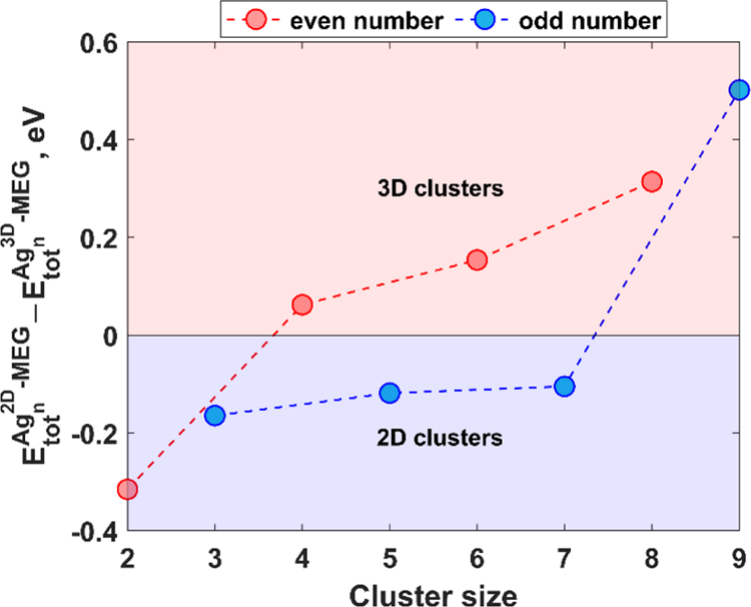
Energy differences between the lowest-energy 2D Ag_*n*_ clusters and lowest-energy 3D clusters (Δ*E*_2D→3D_).

The formation of 3D clusters on weakly interacting substrates cannot be ruled out under realistic conditions.^38−40^ It is important to note that in the case of support-free Ag clusters, 2D-to-3D transition occurs when *n* = 7. More specifically, the pentagonal–bipyramidal structure of the Ag_7_ cluster becomes more stable than the planar structure of Ag_7_.^41^ Similar planar-to-3D transition at *n* = 7 was reported for coronene-supported Ag clusters.^42^ Back to our case, the issue on 2D-to-3D transition is still to be addressed. As noted above, the relatively low barriers for diffusion suggest primarily that being adsorbed onto the MEG surface, Ag atoms tend to form clusters rather than remaining trapped at the initial adsorption sites. However, this knowledge is still insufficient to conclude on the nature of the preferred early-stage nuclei (2D or 3D). Therefore, additional density functional theory (DFT) calculations have been performed to explore the interaction between 3D Ag_*n*_ (*n* = 2–9) clusters and epitaxial graphene on 4H-SiC. The optimized structures of the MEG-supported 3D Ag_*n*_ clusters are shown in Figures S12–S18 (Supporting Information). Analysis of the energy differences between the lowest energy 2D and 3D (Δ*E*_2D→3D_) Ag_*n*_ clusters on MEG (Figure 7, see also Table S1 and Figure S19, Supporting Information) indicates that even-numbered silver clusters with *n* ≥ 4 surfaces favor 3D structures over planar ones at the early stages of growth. Ag_2_ dimer parallel to the MEG surface is a more energetically preferred configuration compared to the structure with perpendicular orientation. Odd-numbered clusters still prefer to form 2D structures up to *n* = 7. Within the considered cluster size range, only the 3D Ag_9_ cluster (Figure S18, Supporting Information) is more stable than the planar counterpart.

### 2.4 Adsorption of CO on MEG-Supported Ag Nanoclusters

Since pseudomorphic silver clusters on MEG are more corrugated/buckled compared to pseudoepitaxial clusters, we have chosen the pseudoepitaxial Ag clusters as models of Ag clusters for further detailed investigation and for designing CO sensors. The most stable systems [Ag_*n*_ (*n* = 1, 3, and 7)/MEG/SiC] were further examined to evaluate the possibility of Ag-nanoparticle-decorated MEG to be used for the chemoresistive sensing of carbon monoxide. Being an odorless and poisonous gas, CO poses a substantial threat to human health.^43^ Therefore, the development of novel sensing materials to design real-time CO detection systems is extremely welcomed. Due to its high conductivity, large surface area, and high signal-to-noise ratio, epitaxial graphene on SiC has recommended itself as an effective electrical transducer for the sensing of toxic heavy metals and hazardous volatile organic compounds.^44−46^ The key principle behind this sensing is that the epitaxial graphene resistance changes upon exposure to liquid or gas and returns to the initial resistance after the removal of the liquid or gas. Since the CO molecule by its properties is physically adsorbed onto bare epitaxial graphene,^47^ it is unlikely to affect measurably the epitaxial graphene resistance. In contrast, the decoration of epitaxial graphene with silver nanoparticles of cluster origin enables introducing additional reactive sites, thereby enhancing the adsorption capability and CO sensing performance. Ideally, high-performance CO sensors must encompass a fast response and a short recovery time. The quick sensor response can be achieved through providing a large degree of charge transfer between CO molecules and Ag-decorated MEG. This will make a substantial change in the electrical resistivity of the epitaxial graphene after CO adsorption if it comes to fruition. The second descriptor of sensor performance—recovery time—is correlated with adsorption energy: the smaller the adsorption energy, the easier the CO desorption. This precondition is of particular importance to ensure the short recovery time.

**Figure 8:**
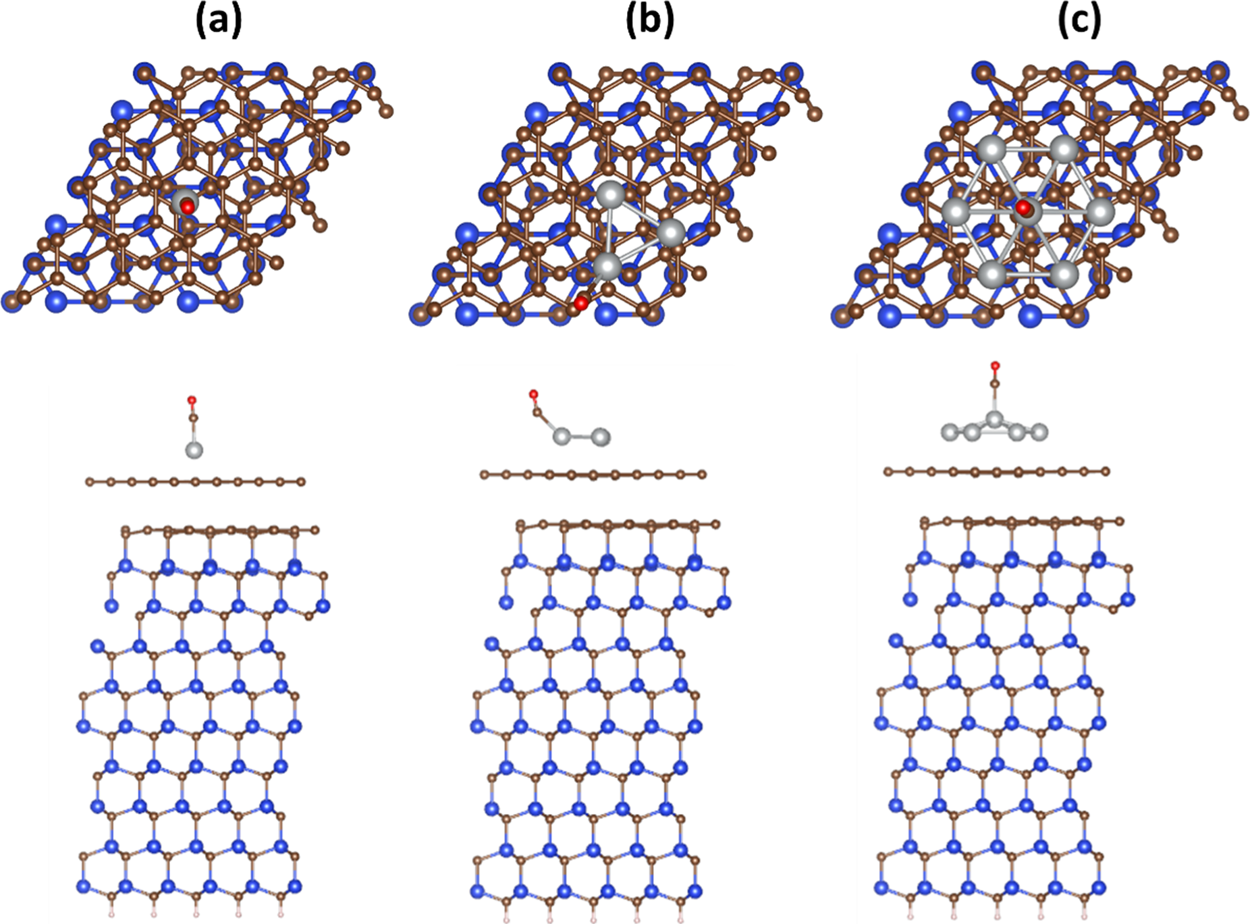
Top and side views of the most stable configuration of (a) Ag_1_/MEG, (b) Ag_3_/MEG, and (c) Ag_7_/MEG systems after CO adsorption. Blue, brown, grayish, and red balls designate Si, C, Ag, and O atoms, respectively.

Figure 8 exhibits the top/side views of Ag_*n*_ (*n* = 1, 3, and 7)/MEG/SiC systems after CO adsorption. CO is preferentially adsorbed vertically above the single Ag_1_ atom and central silver atom of the Ag_7_ cluster, while the adsorbed CO molecule is tilted with respect to the normal to the planar Ag_3_ cluster. The C–O bond distance for the adsorbed CO molecule is slightly elongated compared to that of the isolated CO molecule (1.144 Å) and stretched to 1.152 Å for CO–Ag_7_/MEG (see Table 2). The Ag–C bond length tends to increase with increasing cluster size (Table 2), pointing out a decrease in CO adsorption energy. Indeed, uncorrected [basis set superposition error (BSSE)-corrected] *E*_ads_ is estimated to be 1.506 (0.911), 1.156 (0.515), and 0.794 (0.116) eV for CO adsorption on Ag_1_/MEG, Ag_3_/MEG, and Ag_7_/MEG, respectively. It is obvious that the adsorption energy of CO on Ag_1_ and Ag_3_ is larger than the lower limit of the chemisorption (0.5 eV).

**Table 2:** Parameters Describing the Adsorption of the CO Molecule on Substrate-Supported Silver Nanoclusters^a^

			total charge on CO, e^–^		total charge on Ag_*n*_, e^–^	
structure	Ag–C bond length, Å	C–O bond length, Å	Hirshfeld	Voronoi	Hirshfeld	Voronoi
CO–Ag_1_/MEG	2.071	1.149	0.033	0.013	0.326 (0.297)	0.372 (0.319)
CO–Ag_3_/MEG	2.156	1.151	–0.003	–0.016	0.380 (0.373)	0.422 (0.405)
CO–Ag_7_/MEG	2.224	1.152	–0.023	–0.054	0.348 (0.341)	0.424 (0.387)

a The total charge on Ag_*n*_ clusters before CO adsorption is shown in parentheses.

From an experimental point of view, there is a direct correlation between adsorption energy and sensor recovery time: the larger the adsorption energy, the longer the recovery time. In the case of Ag_3_/MEG and Ag_7_/MEG systems, the adsorption of CO molecules can be referred to weak chemisorption. Thus, relatively short recovery time is anticipated for the proposed sensor configuration. On the other hand, another important factor affecting sensing performance is the charge transfer between the adsorbed CO and the Ag_*n*_/MEG surface since it governs the electrical conductivity of epitaxial graphene and hence sensor response.

**Figure 9:**
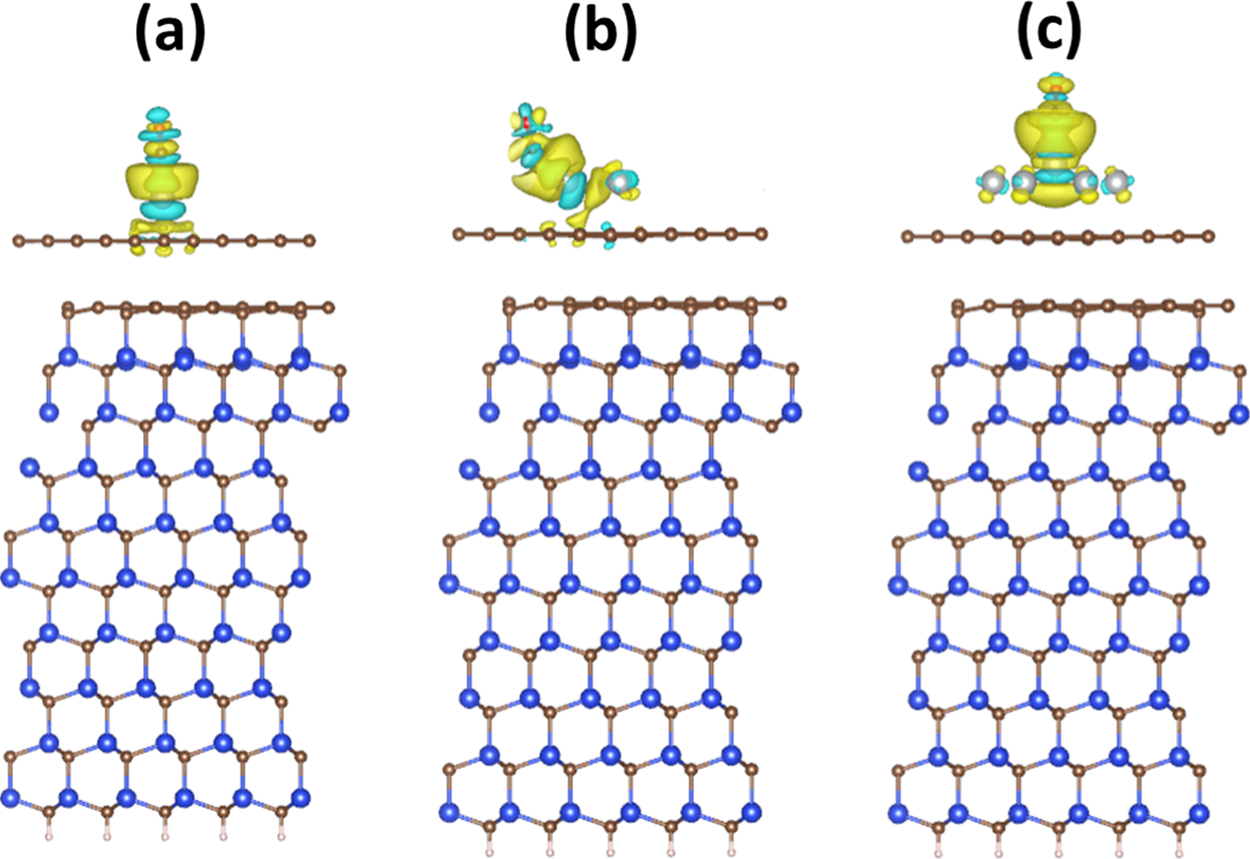
3D CDD plots with an iso-surface value of 0.001 e Å^–3^ for (a) CO–Ag_1_/MEG, (b) CO–Ag_3_/MEG, and (c) CO–Ag_7_/MEG systems. Yellow and cyan colors represent charge accumulation (positive) and depletion (negative) regions, respectively. CDD was calculated by using the following equation: Δρ = ρ_CO–Ag_*n*_/MEG_ – ρ_Ag_*n*_/MEG_ – ρ_CO_.

We revealed that initially the charge transfer occurs from the CO molecule to the silver monomer (Table 2), while reverse charge transfer takes place between CO and larger silver clusters. Since Ag_1_ partly donates its unpaired electron to graphene, it can accept an extra electron from CO, whereas charge redistribution in Ag_3_ and Ag_7_ nanoclusters results in charge accumulation on the CO molecule. Charge density difference (CDD) calculations (Figure 9) additionally corroborate the results of charge population analysis, showing increased charge accumulation on CO adsorbed on larger silver nanoclusters.

The obtained results indicate that only CO adsorption on Ag_1_ and Ag_3_ results in the involvement of graphene in charge redistribution at the interface, while the adsorption of CO on the weakly bonded Ag_7_ cluster does not affect graphene at all. Thus, isolated silver monomers and trimer-based nanoparticles can be regarded as the most effective sensing elements for fast and precise CO detection. Recently, the high affinity of CO to Ag_1_ supported by the Fe_3_O_4_(001) surface has been demonstrated,^48^ which is much larger compared to that of bulk silver and large silver nanoclusters. This phenomenon was explained in terms of d-band theory, according to which the charge transfer between the substrate and Ag_1_ modifies the d-states of silver and strengthens the Ag_1_–CO bond. It was also found that due to the strong Ag_1_–CO bonding, the Ag_1_–support interaction is weakened. In contrast, we revealed that the total charge transferred from Ag_1_ with adsorbed CO to graphene is larger than that from Ag_1_ without adsorbed CO to graphene, indicating the CO-mediated increase of Ag/MEG interaction, in line with the fact that the Ag–graphene distance was reduced from ∼2.08 to ∼2.03 Å to accommodate the CO molecule in the most favorable configuration.

**Figure 10:**
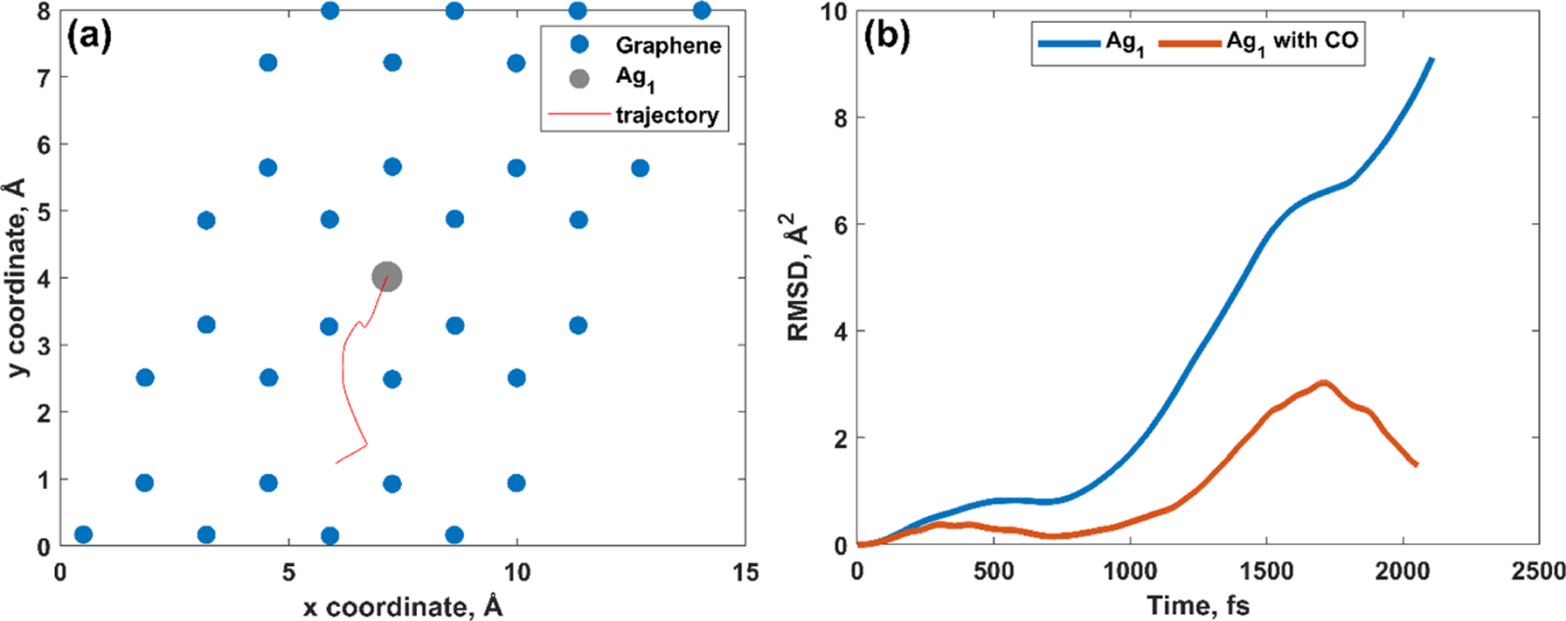
(a) Migration trajectories of the Ag_1_ monomer onto the MEG substrate predicted by MD simulation at 300 K for 2.1 ps and (b) plot of RMSD vs time of Ag_1_ on MEG before and after CO adsorption. Note: RMSD was calculated by using the Einstein equation.^49^

It should be, however, emphasized that the obtained results are valid only at *T* = 0 K, while the room-temperature effects may alter both the cluster stability of selected Ag clusters and the planar-to-3D transition. We, therefore, stress the room-temperature stability of the MEG-supported small-sized silver clusters as another important factor of the efficient CO detection. Indeed, planar silver clusters may migrate on the MEG surface and aggregate to larger clusters (including 3D) upon the introduction of CO under room and moderate temperatures, thereby leading to (i) a dramatic decrease of the active surface atoms, (ii) a redistribution of the interfacial charge transfer due to weakening of the Ag/MEG interaction, and (iii) hence an instability of the whole Ag/MEG system under realistic environmental conditions. Molecular dynamics (MD) calculations enabled us to better comprehend the room-temperature stability of three selected clusters before and after CO adsorption: Ag_1_, Ag_3_, and Ag_7_, respectively. At room temperature, the Ag_1_ monomer that initially occupied the hollow site moves away from its preferred adsorption position (Figure 10a), freely migrating over the MEG surface. This is also evidenced by the analysis of the time dependence of the root-mean-square displacement (RMSD) for a short period (Figure 10b).

**Figure 11:**
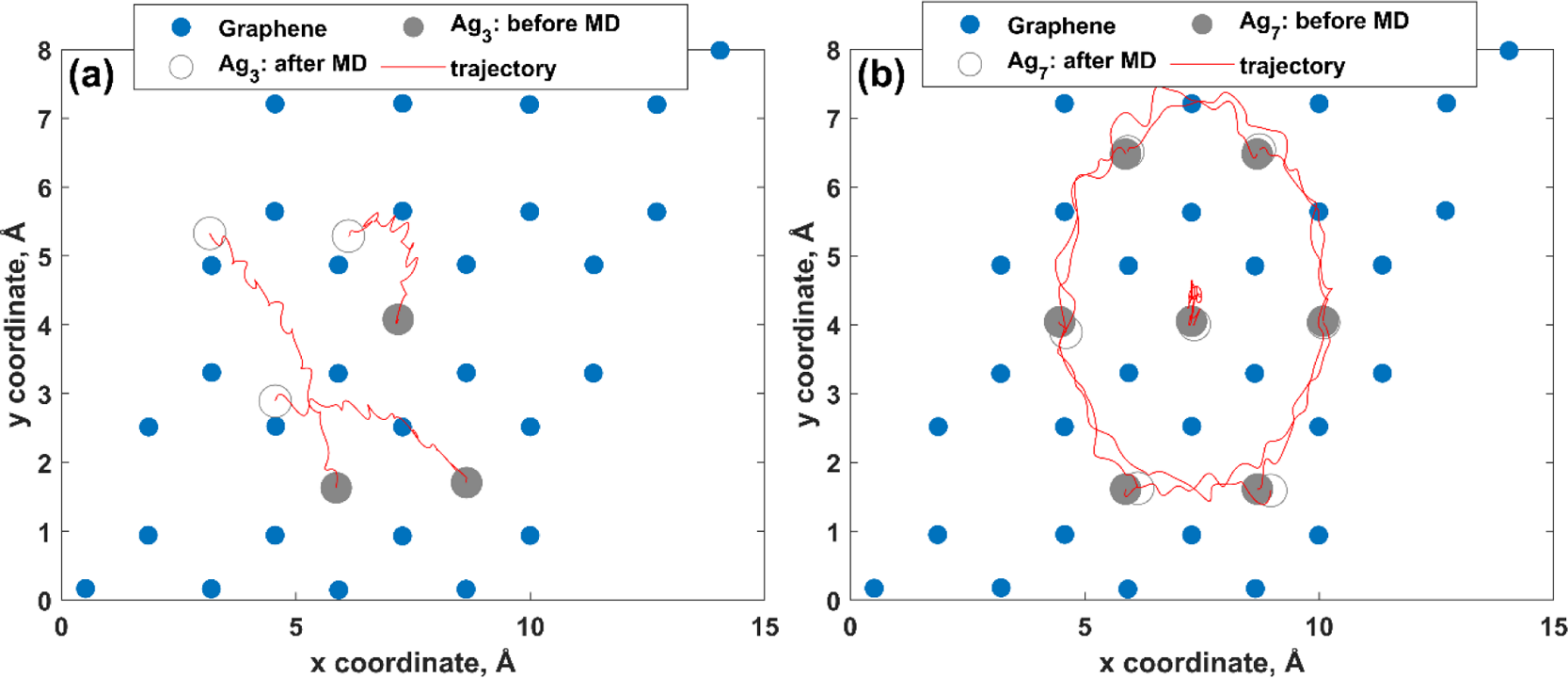
Migration trajectories of (a) Ag_3_ and (b) Ag_7_ clusters onto the MEG substrate predicted by MD simulations at 300 K for 2.3 ps.

It is interesting to note that the interaction between the CO molecule and MEG-supported Ag_1_ monomer suppresses the mobility of the single metal atom that is confirmed by reduced RMSD compared to CO-free Ag_1_ monomer (Figure 10b, see also Figure S20, Supporting Information). Based on the analysis of room-temperature fluctuations of Ag_1_–CO bond length (Figure S21, Supporting Information), we defined the average value of 2.14 ± 0.07 Å, which is larger than the value of 2.071 Å that is predicted by 0 K DFT calculations. It was also found that despite the collective motion of silver atoms and their displacements from the equilibrium positions (Figure S22, Supporting Information), Ag_3_ and Ag_7_ clusters preserve their planar structure at room temperature (Figures 11 and S23, Supporting Information). It is worth noting that the diffusion of silver atoms belonging to the Ag_7_ cluster occurs through rotation around the *z* axis (note: the central atom of the Ag_7_ cluster weakly fluctuates around its equilibrium position), while the Ag_3_ cluster migrates through in-plane translation with partial rotation. Notably, in the presence of a gas molecule, the Ag_3_ cluster still prefers the planar structure (Figure S24a). After 2 ps, the CO–Ag_3_ bond length reaches the value of 2.11 Å at 300 K, which is in good agreement with the 0 K DFT-derived value of 2.15 Å. Concomitantly, we observed CO-mediated reshaping of the Ag_7_ cluster at room temperature from planar to 3D (Figure S24b). This process may result in the partial deactivation of catalytically active metal sites of the Ag_7_ cluster and hence a decrease in the sensing performance.

Considering the above, it seems reasonable to assume that the reliable stabilization strategy for Ag clusters on MEG should be implemented to design the high-performance sensor with reproducible characteristics. Intuitively, such a strategy should be to suppress the surface mobility of Ag clusters though providing stronger metal–support interaction. This will enable the small silver clusters to be trapped and the whole sensor architecture to be stabilized. In the case of the Ag/MEG system, this can be realized in several ways, including defect engineering, strain engineering, electronic doping of graphene, and so forth. To start with, here, we investigate the early stages of silver clustering on ideal defect-free MEG on 4H-SiC. With no reliable theoretical data available on the interaction between Ag clusters and defectless MEG, the current results will ensure the required scientific background for further investigation of the Ag/MEG system with optimized sensing characteristics toward high-performance CO detection.

## 3 Conclusions

The early-stage nucleation of nanosized Ag_*n*_ clusters with *n* = 1–9 on MEG was systematically studied by using DFT and MD methods, mainly focusing on changes of the metal–support interaction with cluster size increase with the aim to understand eventual nanoparticle formation. By investigating the diffusion over the MEG surface, we found that the Ag adatom prefers to sit at the hollow site position of MEG, while there is an energy barrier, ranging within 79–157 meV, to overcome the top site position. We also defined the critical Ag–Ag distance of 5.19 Å at which the attractive metal–metal forces become dominant over metal–support interaction, while the charge transfer from Ag to MEG drops down. It was shown that the pseudoepitaxial growth regime dominates the formation of planar silver clusters. The adsorption and nucleation energies of Ag clusters on MEG exhibit prominent odd–even oscillations, indicating that the adsorption and nucleation of odd-numbered Ag_*n*_ clusters are energetically more favorable processes than those of even-numbered clusters. Due to the strongest interaction with the MEG surface, the Ag_3_ trimer is found to be the most stable early-stage nucleus at MEG for the subsequent growth of Ag nanoparticles. The estimated relationship between Ag–Ag interaction (cohesive energy) and the Ag/MEG interaction (adsorption energy) for all considered clusters indicates that the structural evolution of Ag clusters obeys the island growth mechanism. Last, we note that there is a fundamental correlation between Ag/MEG interaction and Ag–Ag interaction. Particularly, the increase in cluster size leads to an increase in cohesive energy for the Ag_*n*_ cluster and weakens the interaction between Ag adatoms and the MEG surface and local charge transfer from Ag to MEG. The unexpected finding is that the 2D–3D structure transition occurs at *n* = 4 and *n* = 9 for even-numbered and odd-numbered MEG-supported silver clusters, respectively. MD calculations allowed us to get insights into the room-temperature stability of selected odd-numbered silver clusters. MEG-supported isolated Ag_1_ and Ag_3_ nanoclusters are identified as possible sensitive elements for CO detection. The present results gain beneficial insights into the nature of the early-stage nucleation and stability of planar silver nanoclusters on MEG and could help to rationalize the design of the Ag/MEG nanoarchitecture with desired properties toward and to boost the development of high-performance CO sensors.

## 4 Theoretical Methods

To elucidate the main features of Ag adsorption, diffusion, and nucleation on the MEG surface, comprehensive theoretical calculations by using the DFT method were performed. A 4 × 4 slab composed of a honeycomb array of carbon atoms accommodated on top of the reconstructed surface of 4H-SiC(0001) was chosen as a model of the MEG/4H-SiC stack (Figure S25, Supporting Information). According to this model, the bottom carbon atoms with dangling bonds are saturated by hydrogen, while the buffer layer is partly covalently bonded to Si surface atoms to saturate the interfacial dangling bonds.

To investigate the diffusion barriers for Ag and to predict the most favorable adsorption sites, we mimic the Ag migration phenomenon at the MEG surface by using the following scheme: (1) we first located the Ag atom at the top site (above the C atom) in the beginning of the diffusion path → (2) we then performed geometrical optimization of the system with constraints to Ag movements in *x* and *y* directions → (3) we finally displaced the Ag adatom to the next point along the diffusion path accompanied by relaxation only along the *z* direction. The migration of the Ag atom along two paths will be examined as follows: (i) toward an empty site (without another preadsorbed Ag atom) and (ii) toward an occupied site (with preadsorbed Ag atom).

All ab initio calculations were performed by using the SIESTA code^50^ including the van der Waals density functional proposed by Berland and Hyldgaard.^51^ The construction of pseudopotentials for H, Si, C, O, and Ag was carried out within the Troullier–Martins scheme by using the ATOM code.^52^ Along with the elements involved in the studied material system, we also consider oxygen as being a part of CO which is an example of detected analyte. We used a vacuum space of 20 Å along the *z* direction to avoid undesired interactions with neighboring unit cells. The double-zeta polarized basis set with an energy shift of 200 meV was utilized. The Cartesian coordinates of the considered structures were relaxed until the force on each atom reached less than 0.02 eV/Å. A Monkhorst–Pack *k*-point 3 × 3 × 1 mesh was used to sample the Brillouin zone during the optimization process. The mesh cutoff was set at 400 Ry. The charges of Ag adatoms were calculated by Hirshfeld^53^ and Voronoi^54^ population analysis schemes, respectively.

To discriminate the Ag_*n*_/MEG and Ag–Ag interaction, we estimated two types of adsorption energies by using the following relationships^55^
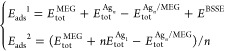
2where *E*_tot_^MEG^ is the total energy of isolated MEG,  is the total energy of isolated Ag_*n*_ cluster, *n* is the number of silver atoms belonging to the cluster,  is the total energy of isolated single silver atom, and  is the total energy of the Ag_*n*_ cluster adsorbed on the surface of the MEG. *E*^BSSE^ is BSSE correction,^56^ which can be defined as
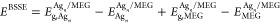
3where  and  are the total energies of the Ag_*n*_ cluster and MEG substrate in the relaxed geometry of the Ag_*n*_/MEG system, while  and  are the total energies of the Ag_*n*_ cluster and MEG substrate in the relaxed geometry of the Ag_*n*_/MEG system by using ghost atoms. The equation for estimation of *E*_ads_^2^ can be easily rearranged as
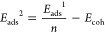
4where *E*_coh_ is the cohesive energy, which can be estimated by using the following expression
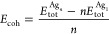
5

Cohesive energy that is determined by eq 5 will be always negative. The more negative the cohesive energy, the more the energy is required to sublimate the Ag_*n*_ cluster. Notably, *E*_ads_^1^ energy exceptionally reflects the interaction between the MEG and Ag_*n*_ clusters, while *E*_ads_^2^ contains two components: cohesive energy of the Ag_*n*_ cluster and adsorption energy *E*_ads_^1^. According to such definitions, *E*_ads_^2^ gives a critical knowledge on the stability of the different clusters on MEG and Ag–Ag interaction strength. By using such definitions for adsorption energies, we argue that the positive values of the adsorption energies imply that the adsorption process is thermodynamically favorable (exothermic process).

To quantify the deformation degree in the Ag_*n*_/MEG systems induced by adsorption events, we calculated the energies associated with the structural changes of Ag clusters and MEG by the following equations
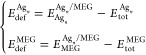
6

Last, we estimated the nucleation energy of Ag clusters on MEG by using the following equation^57^
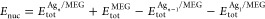
7where the meaning of  and *E*_tot_^MEG^ is the same as that in eq 2, while  and  are the energies of the single Ag_1_ atom and Ag_*n*–1_ cluster adsorbed on MEG. According to such definitions, the positive value of the nucleation energy means that the nucleation process on the surface is energetically unfavorable and vice versa.

MD calculations have been carried out by means of the thermostat implemented into the SIESTA code in the *anneal* regime to investigate the room-temperature (300 K) stability of selected silver planar clusters (Ag_1_, Ag_3_, and Ag_7_) that are considered as possible sensing elements for CO detection. For the sake of saving computational time, a thinner 4 × 4 graphene slab on SiC with two Si–C bilayers (Figure S26, Supporting Information) was used to investigate the time evolution of the position of Ag atoms on the MEG surface and silver cluster structure. Prior to MD calculations, the geometrical optimization of all structures of interest has been performed. A time step and a relaxation time for MD simulations were set to 1 and 25 fs, respectively.

## Supporting Information Available

4 × 4 supercell of MEG; diffusion paths; optimized structures of planar silver clusters on MEG (pseudomorphy and pseudoepitaxy); optimized structures of support-free Ag clusters; charge population analysis; optimized structure of 3D silver clusters; and results of MD calculations (PDF)
