# Supplementary material for: Computational Appraisal of Silver Nanocluster Evolution on Epitaxial Graphene: Implications for CO Sensing
Source: ACS Omega. 2021 Sep 15;6(38):24739–51. doi: 10.1021/acsomega.1c03577 (PMC8482456; doi:10.1021/acsomega.1c03577)
Supplement: Supplementary file 1 — ao1c03577_si_001.pdf [file ao1c03577_si_001.pdf]

Supporting Information
for
Computational appraisal of silver nano-clusters evolution on epitaxial graphene: implications for CO sensing

Ivan Shtepliuk and Rositsa Yakimova

Department of Physics, Chemistry and Biology-IFM, Linköpings universitet, 58183 Linköping, Sweden

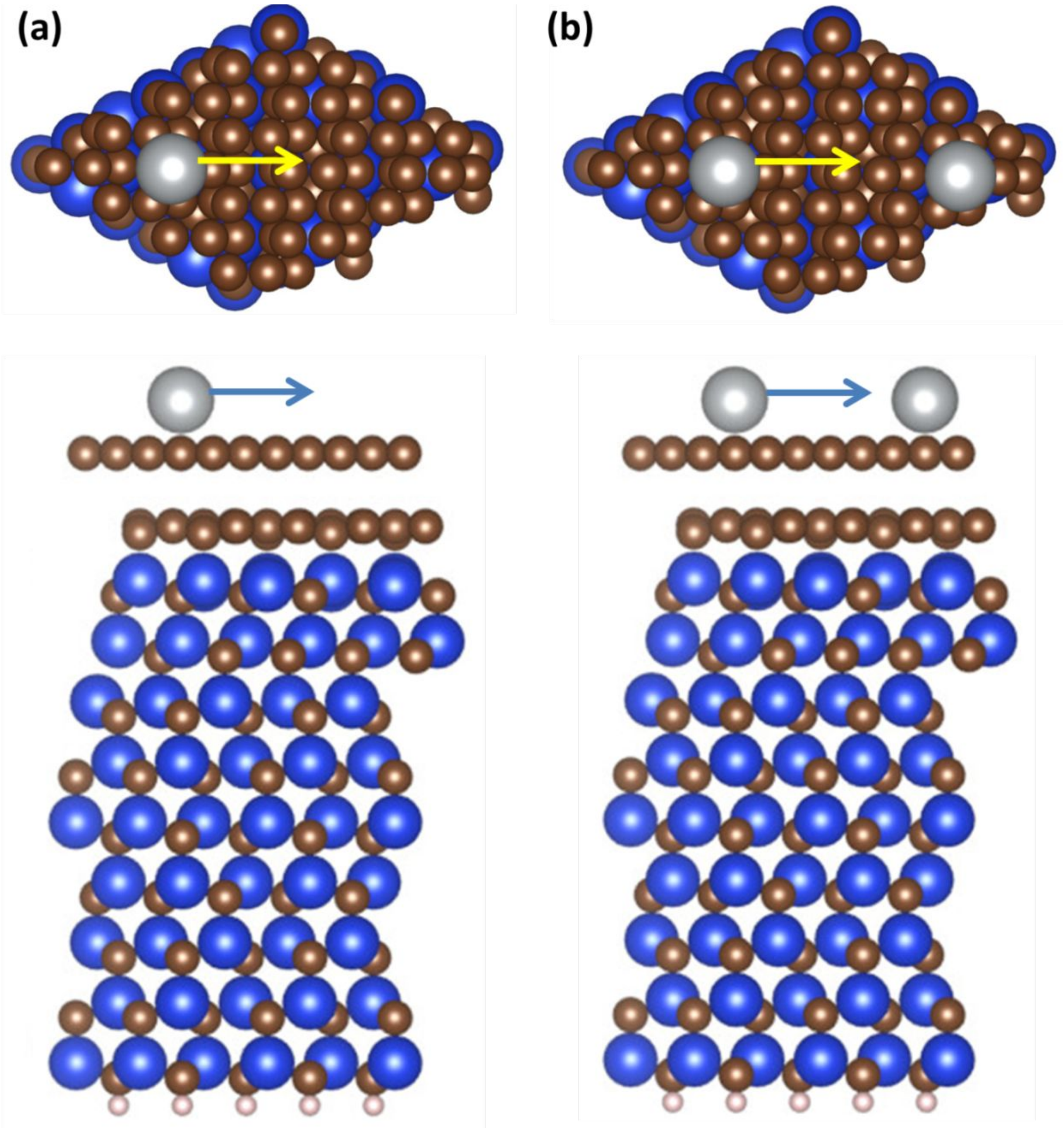

Figure S1. (Top and side views) Diffusion paths over the surfaces of monolayer epitaxial graphene (a) in the absence and (b) in the presence of the already adsorbed Ag adatom.

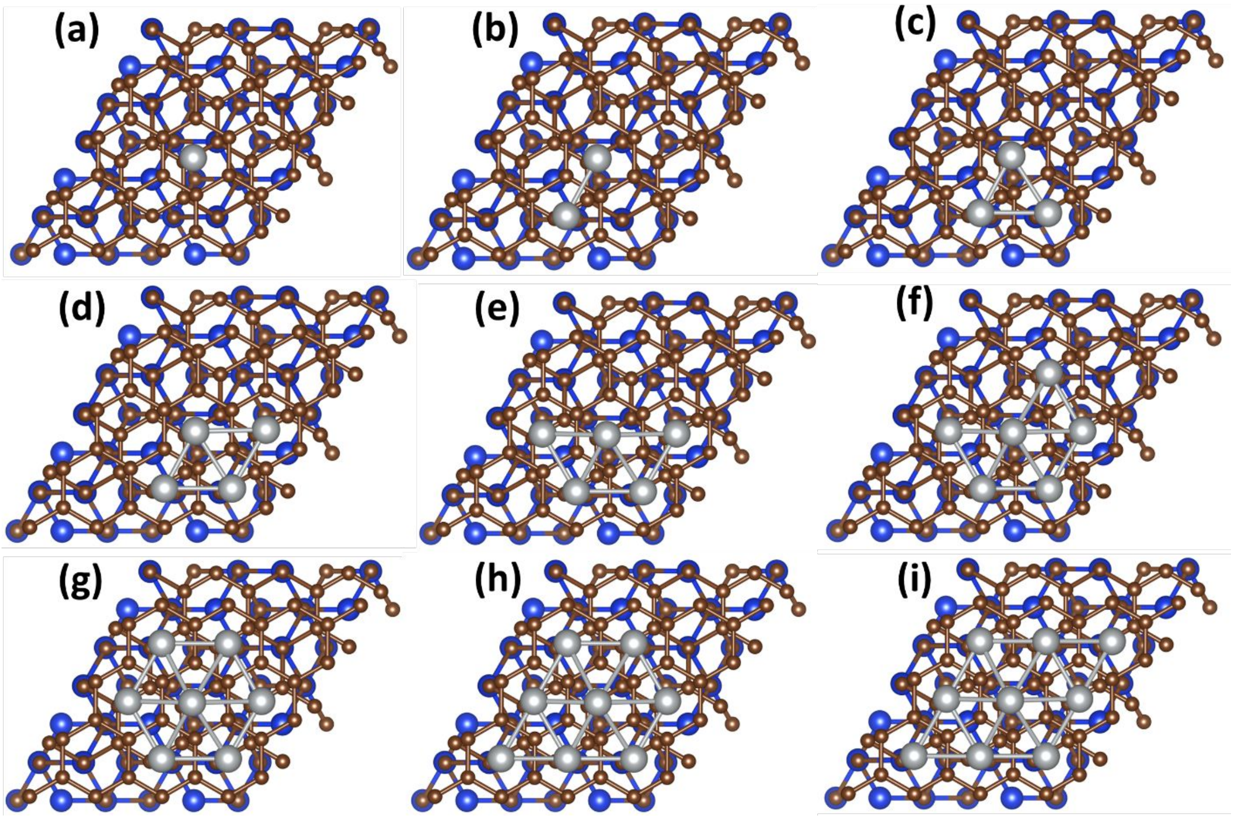

Figure S2. (Top view) The optimized geometrical structures of the monolayer epitaxial graphene after adsorption of different number of Ag atoms: from 1 (a) to 9 (i). Such structures are related to pseudomorphy at hollow sites.

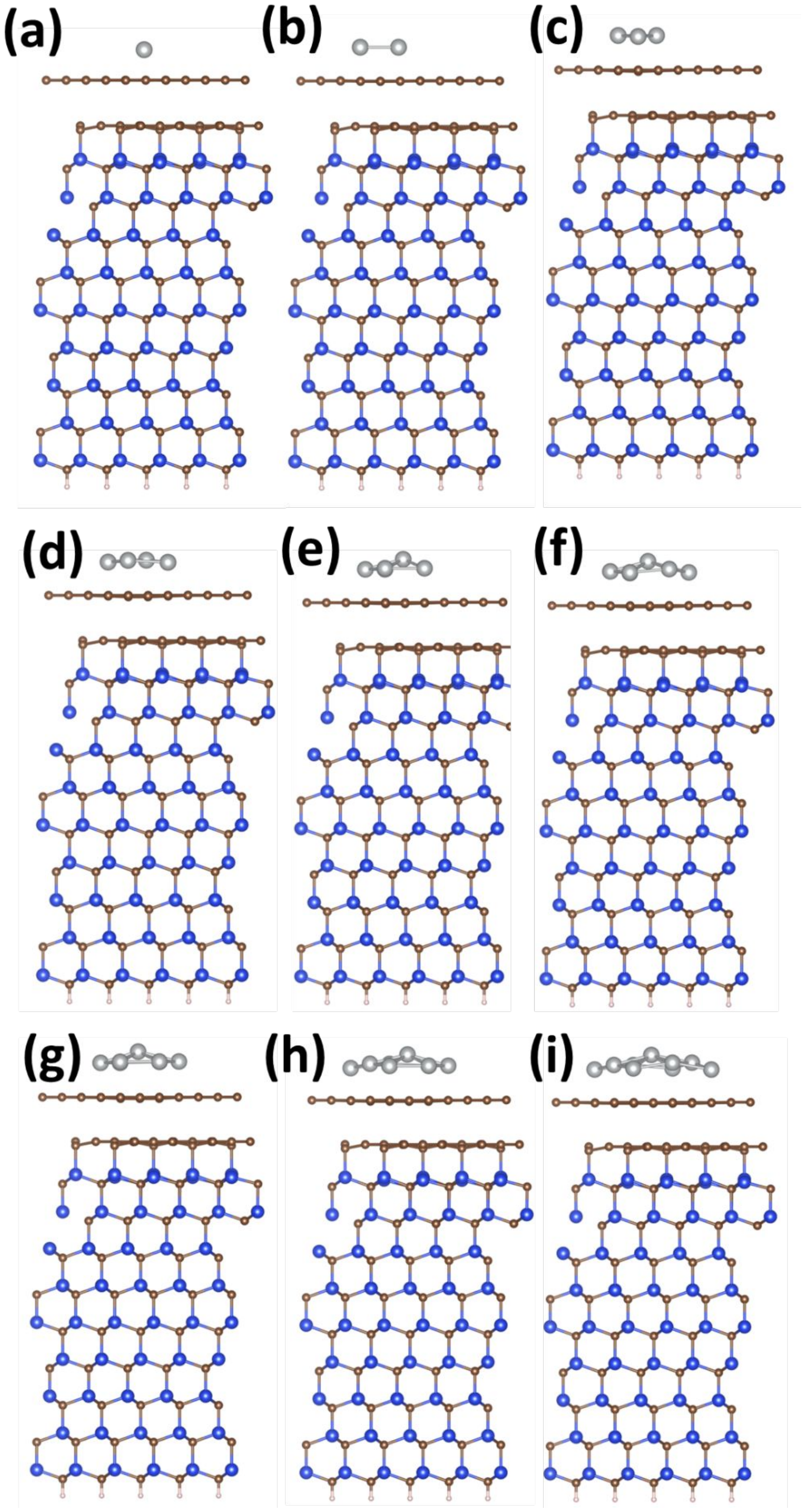

Figure S3. (Side view) The optimized geometrical structures of the monolayer epitaxial graphene after adsorption of different number of Ag atoms: from 1 (a) to 9 (i). Such structures are related to pseudomorphy at hollow sites.

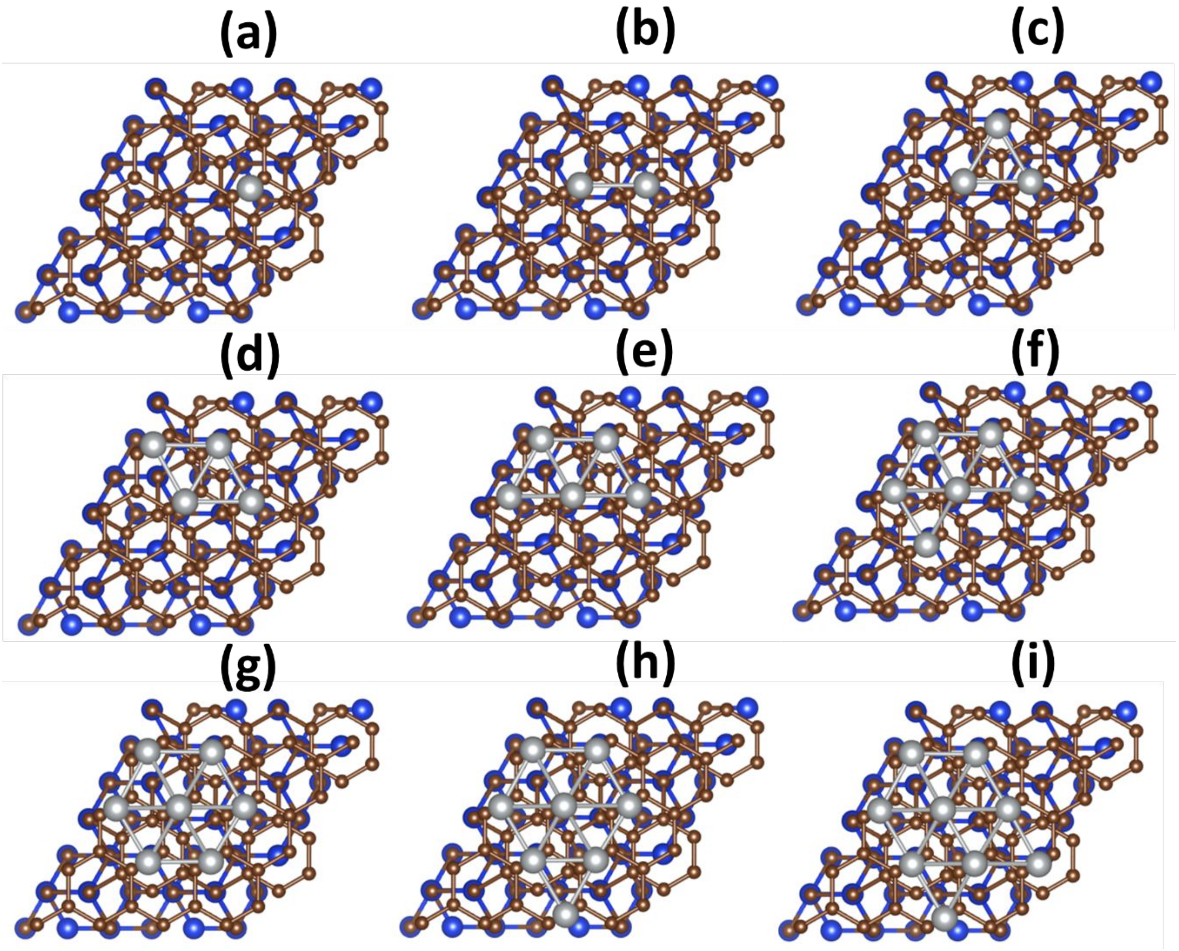

Figure S4. (Top view) The optimized geometrical structures of the monolayer epitaxial graphene after adsorption of different number of Ag atoms: from 1 (a) to 9 (i). Such structures are related to pseudomorphy at bridge sites.

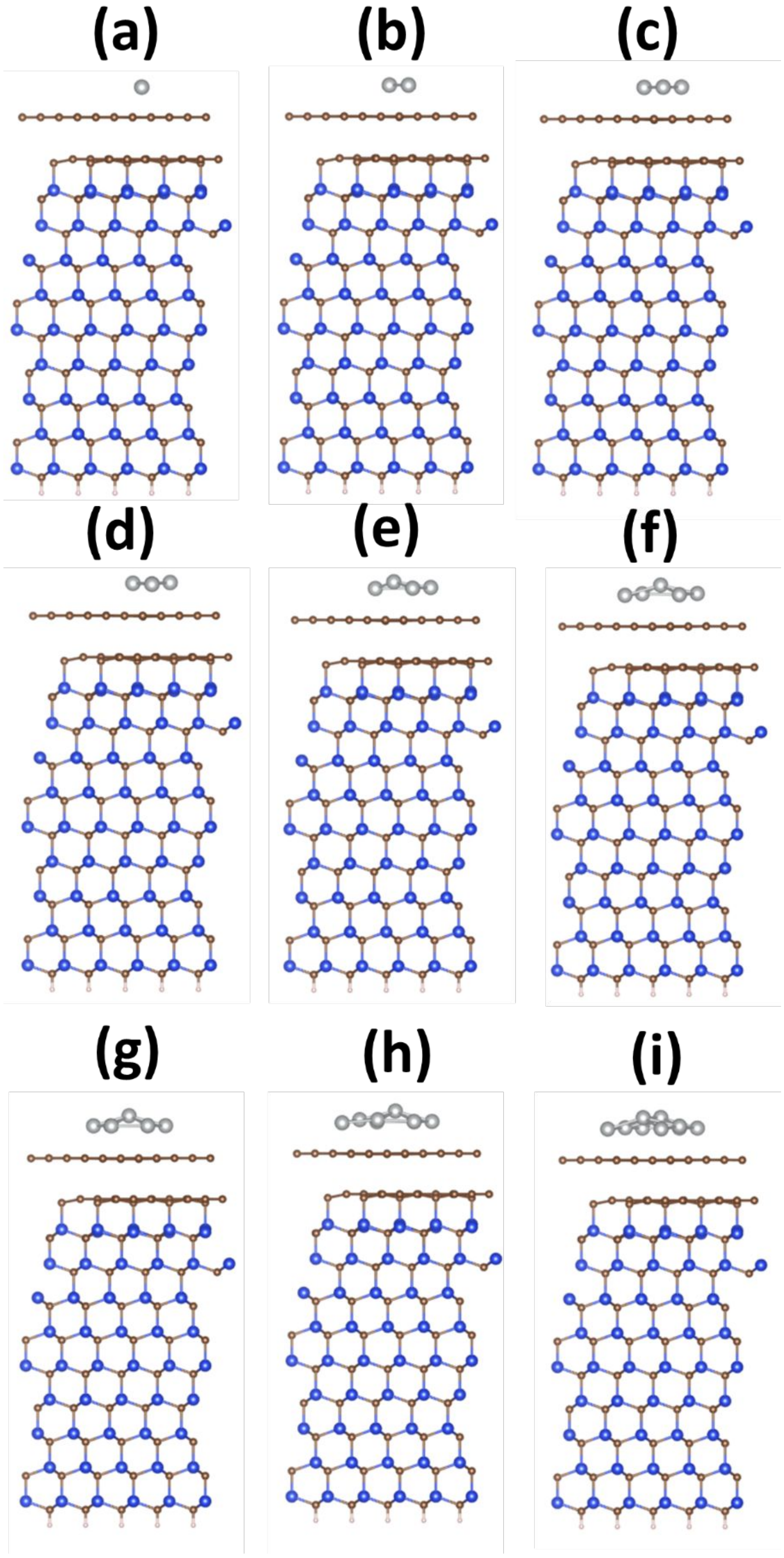

Figure S5. (Side view) The optimized geometrical structures of the monolayer epitaxial graphene after adsorption of different number of Ag atoms: from 1 (a) to 9 (i). Such structures are related to pseudomorphy at bridge sites.

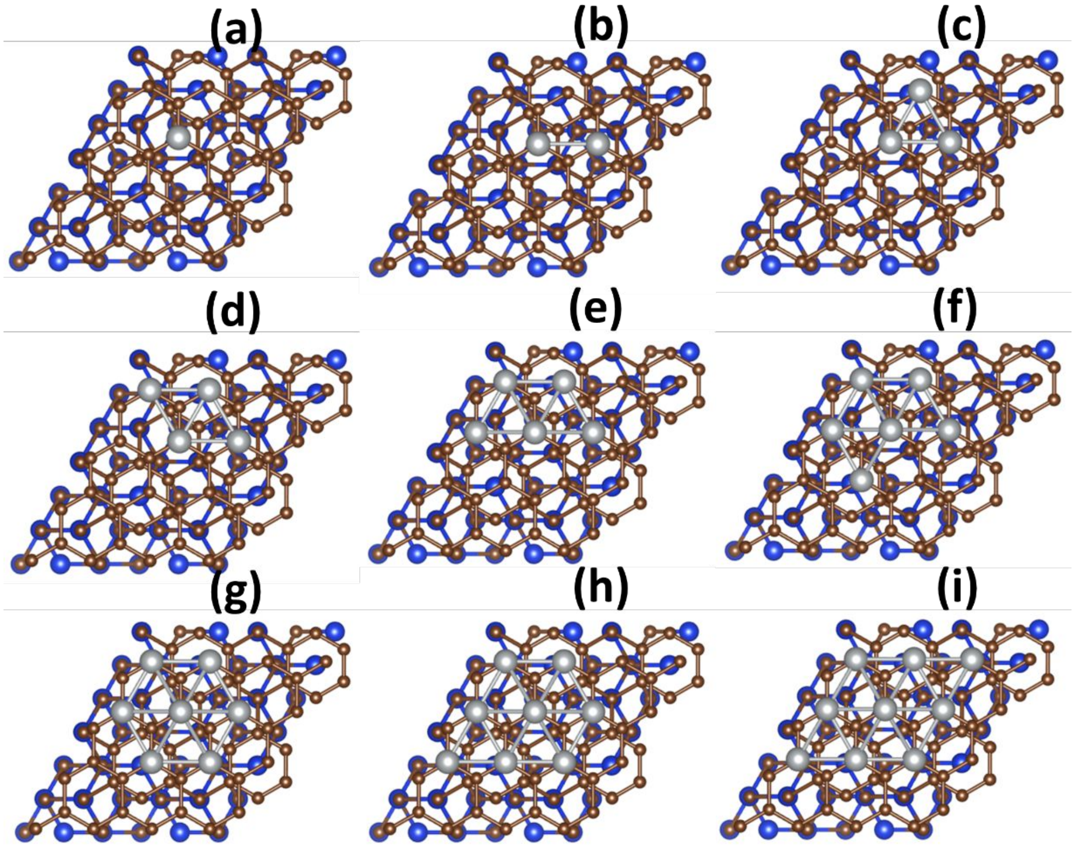

Figure S6. (Top view) The optimized geometrical structures of the monolayer epitaxial graphene after adsorption of different number of Ag atoms: from 1 (a) to 9 (i). Such structures are related to pseudomorphy at top sites.

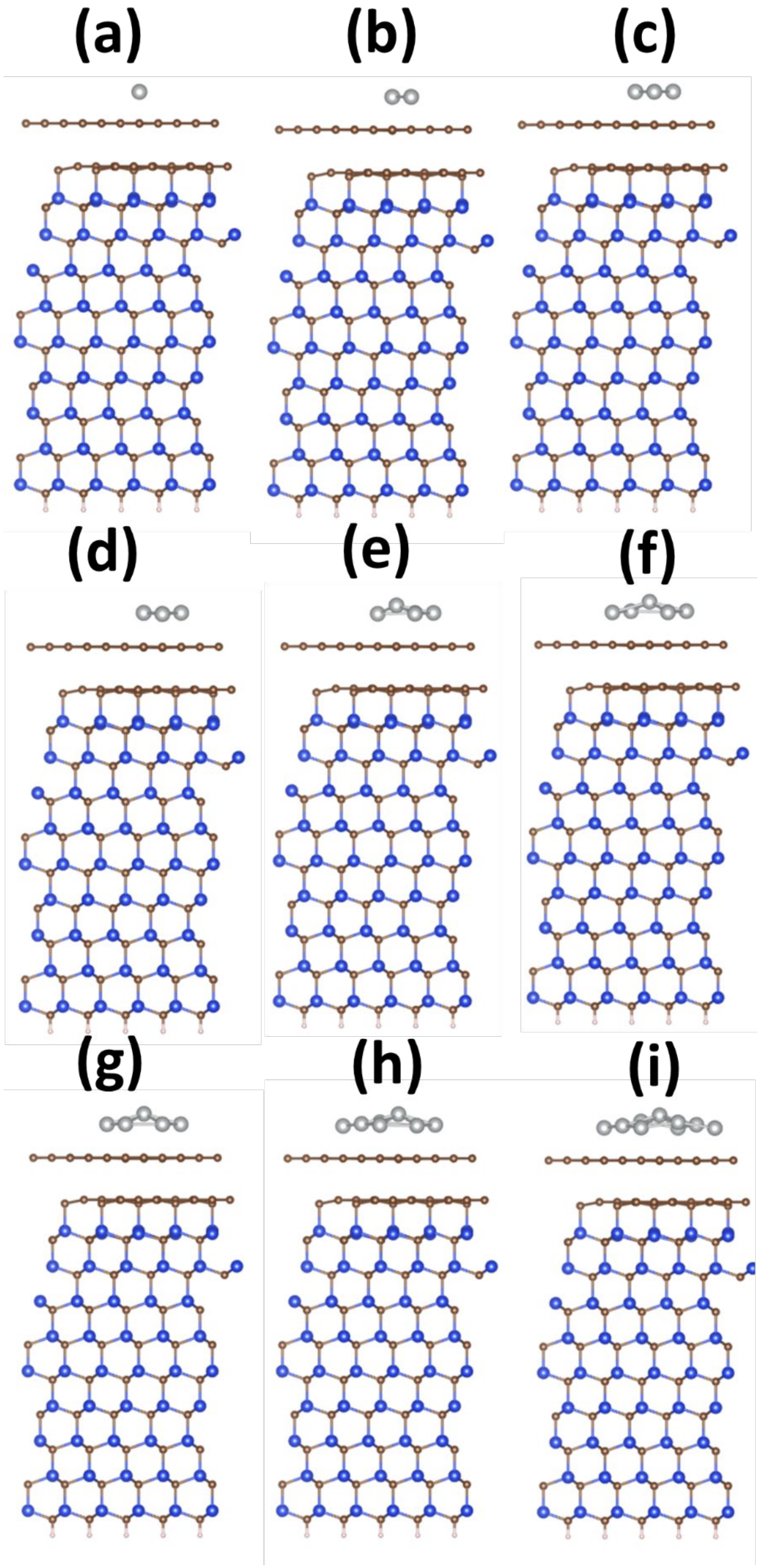

Figure S7. (Side view) The optimized geometrical structures of the monolayer epitaxial graphene after adsorption of different number of Ag atoms: from 1 (a) to 9 (i). Such structures are related to pseudomorphy at top sites.

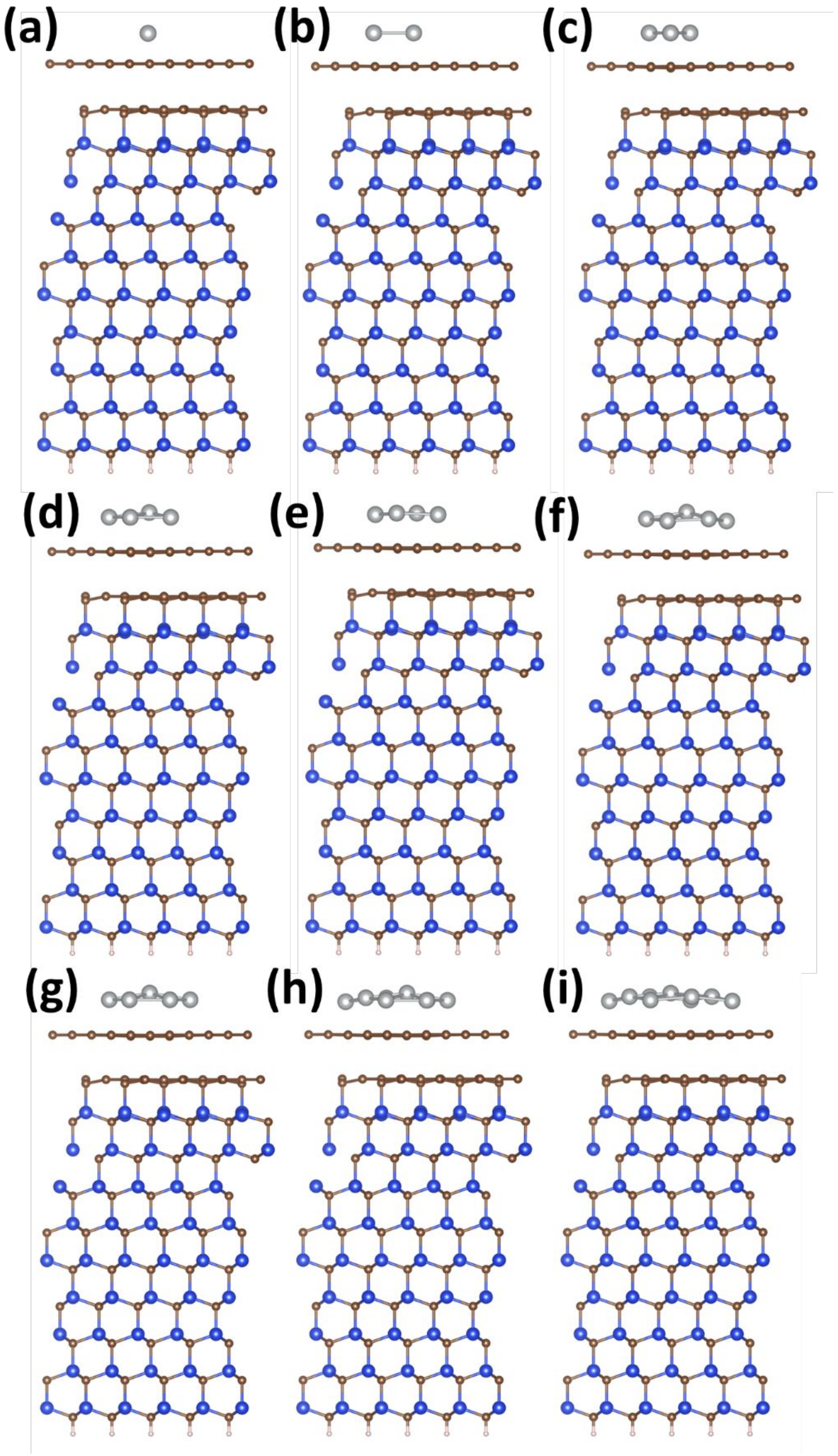

Figure S8. (Side view) The optimized geometrical structures of two-dimensional Ag_n clusters supported by monolayer epitaxial graphene: from Ag_1 (a) to Ag_9 (i), respectively. Such geometrical configuration is assigned to the case of pseudoepitaxy. Blue, brown, and greyish balls designate Si, C and Ag atoms, respectively.

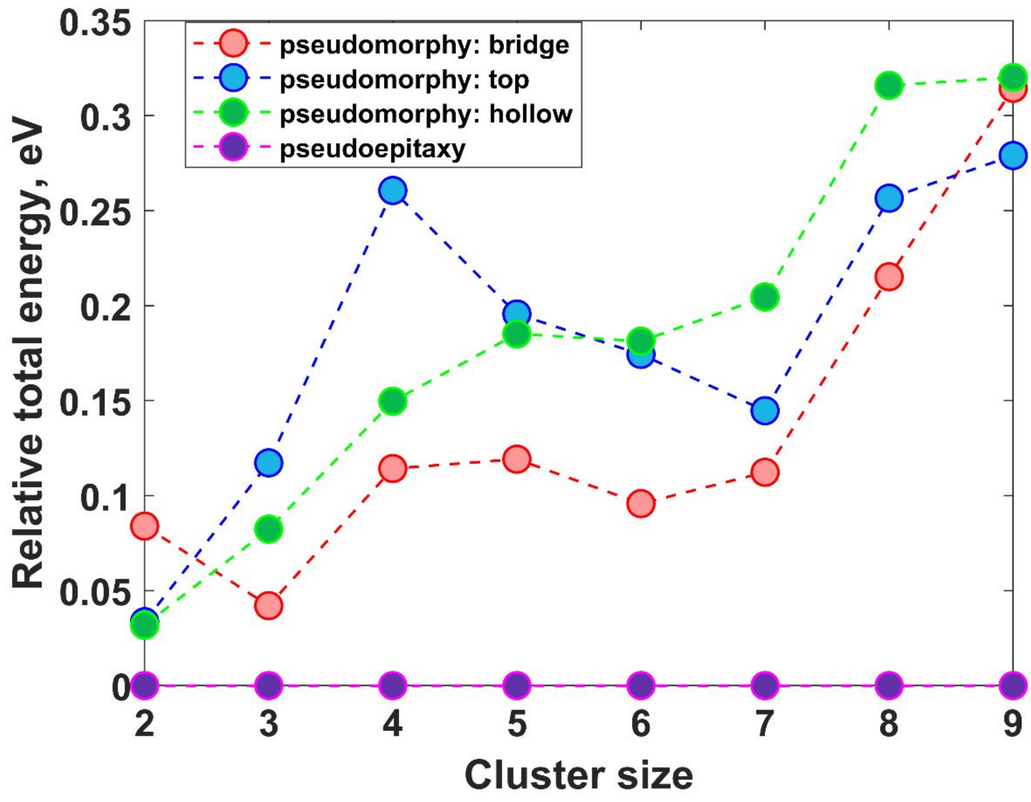

Figure S9. Dependence of the relative total energy of Ag_n/MEG system on the cluster size *n* for pseudomorphy and pseudoepitaxy cases. All values of total energy were normalized to the most negative total energy, i.e. total energy of pseudoepitaxial structures.

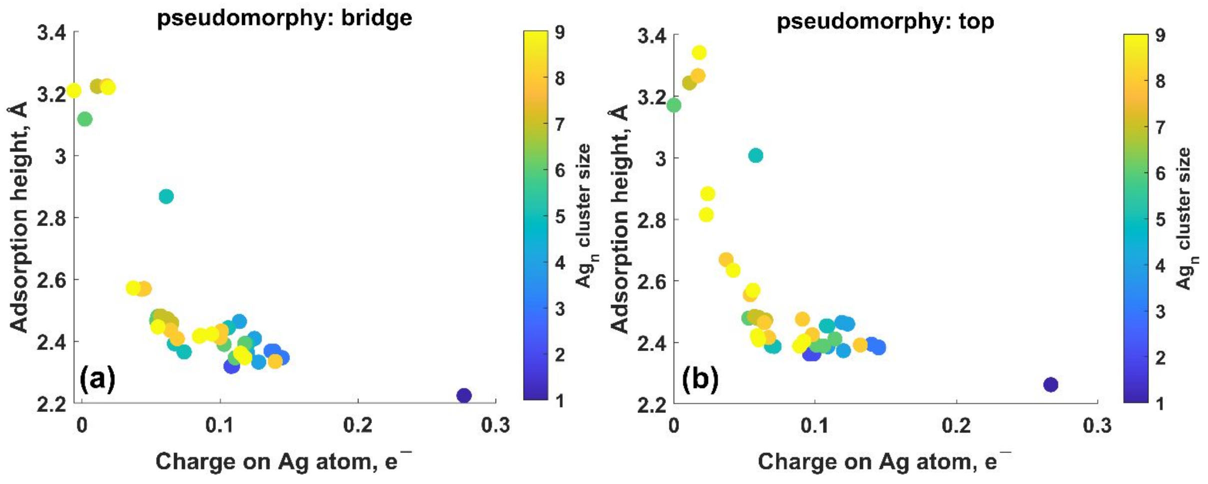

Figure S10. Correlation between adsorption height of Ag adatom in Ag_n cluster and effective charge on the same atom: (c) pseudomorphy at bridge sites and (d) pseudomorphy at top sites respectively.

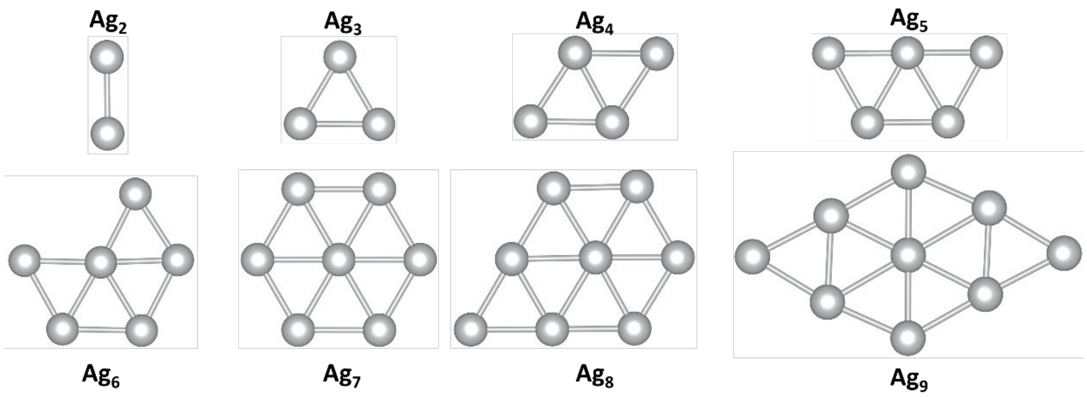

Figure S11. (Top view) The optimized geometrical structures of two-dimensional free-standing (support-free) Ag_n: from Ag_2 to Ag_9, respectively.

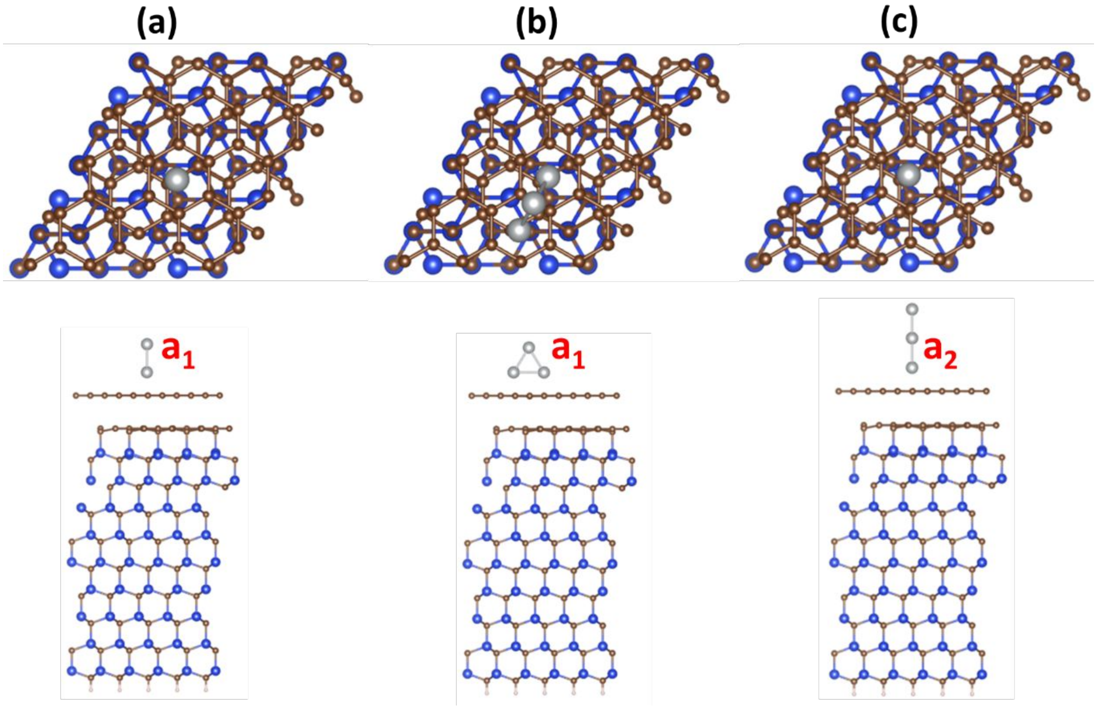

Figure S12. (Top and side views**)** The optimized geometrical structures of the monolayer epitaxial graphene after adsorption of non-planar Ag_2 (a) and Ag_3 (b, c) clusters. a_i designates the cluster structure.

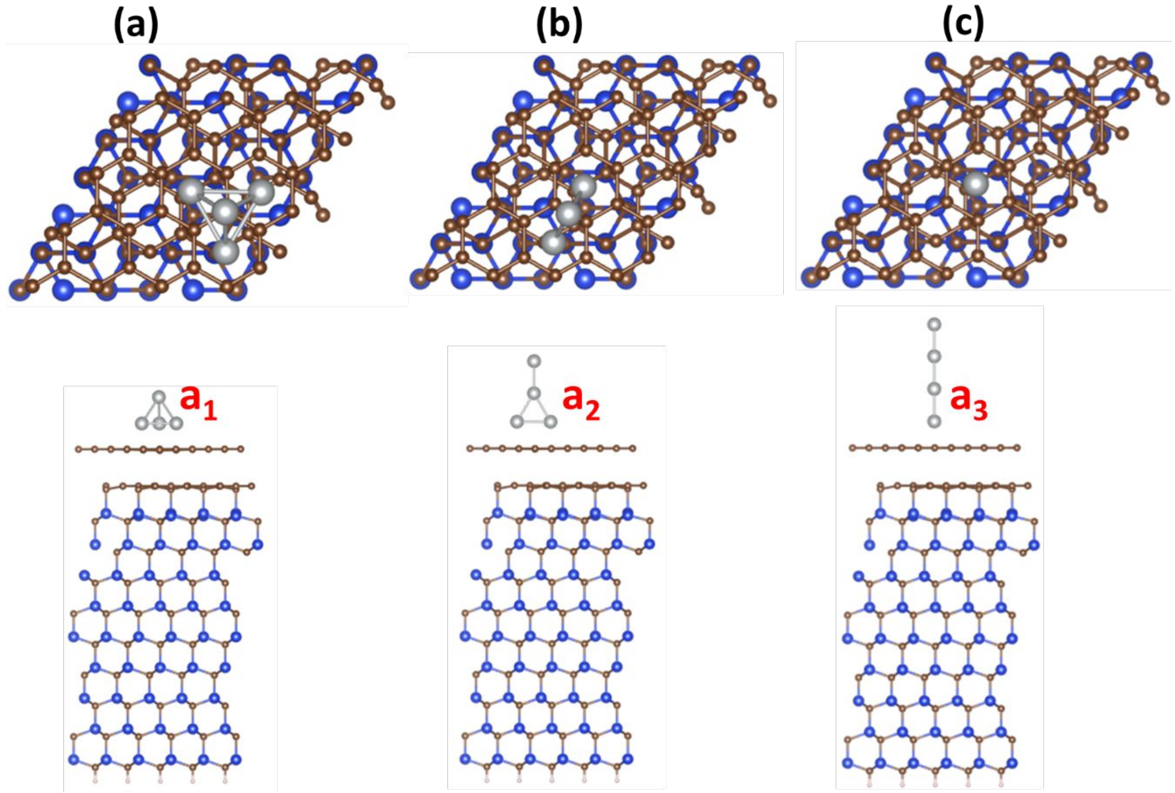

Figure S13. (Top and side views**)** The optimized geometrical structures of the monolayer epitaxial graphene after adsorption of non-planar Ag_4 clusters (a-c). a_i designates the cluster structure.

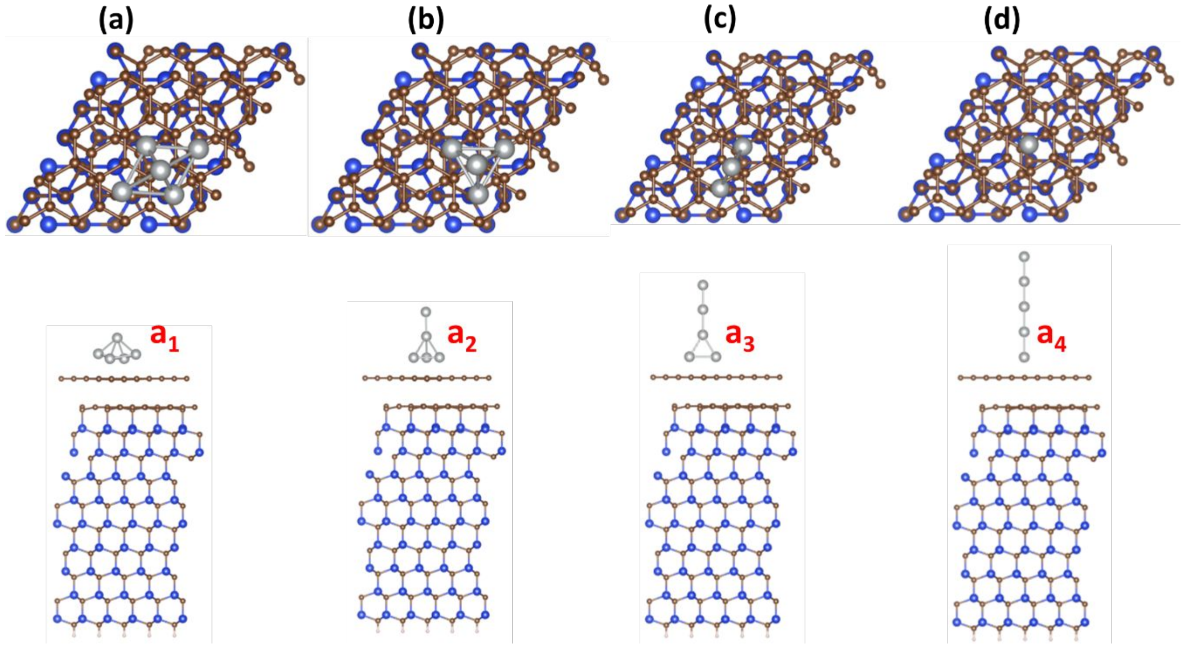

Figure S14. (Top and side views**)** The optimized geometrical structures of the monolayer epitaxial graphene after adsorption of non-planar Ag_5 clusters (a-d). a_i designates the cluster structure.

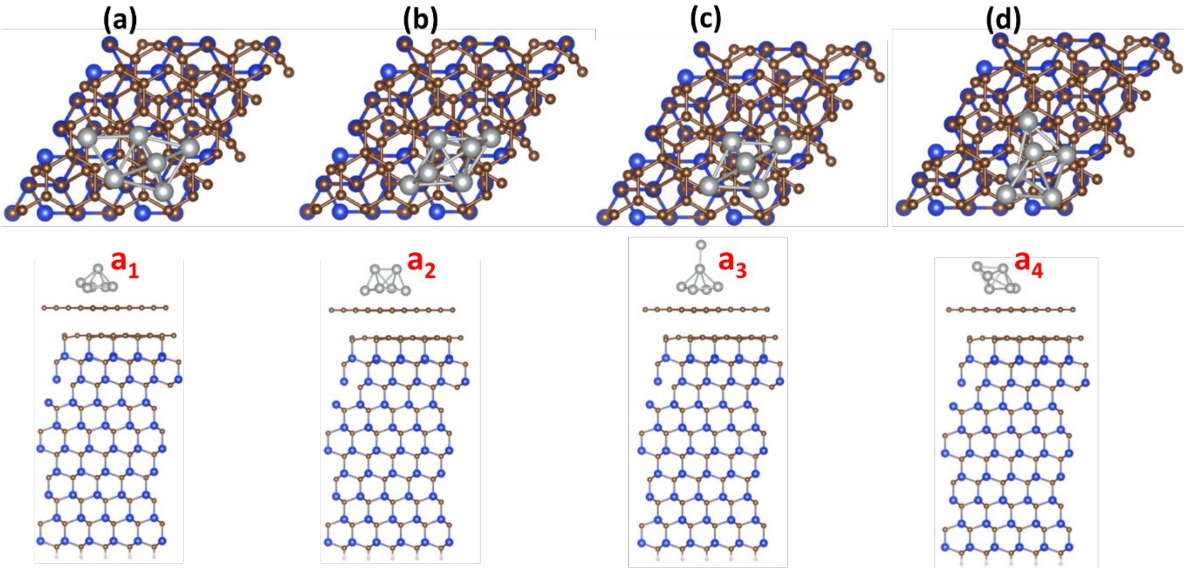

Figure S15. (Top and side views**)** The optimized geometrical structures of the monolayer epitaxial graphene after adsorption of non-planar Ag_6 clusters (a-d). a_i designates the cluster structure.

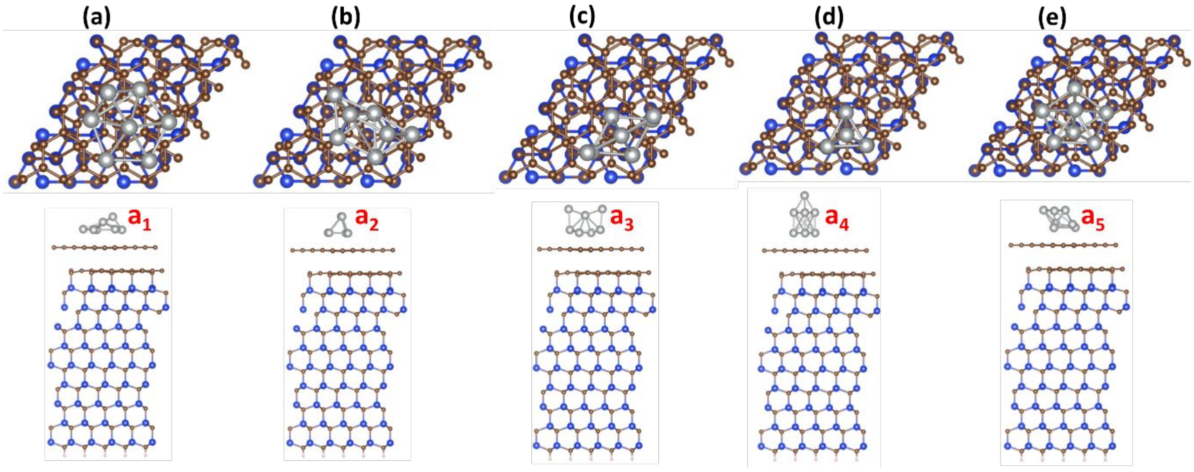

Figure S16. (Top and side views**)** The optimized geometrical structures of the monolayer epitaxial graphene after adsorption of non-planar Ag_7 clusters (a-e). a_i designates the cluster structure.

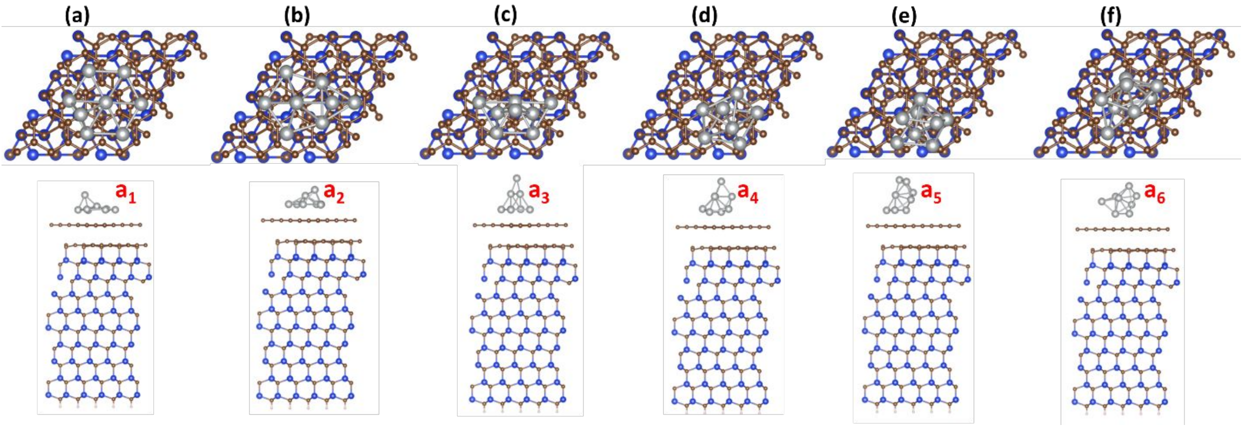

Figure S17. (Top and side views) The optimized geometrical structures of the monolayer epitaxial graphene after adsorption of non-planar Ag_8 clusters (a-f). a_i designates the cluster structure.

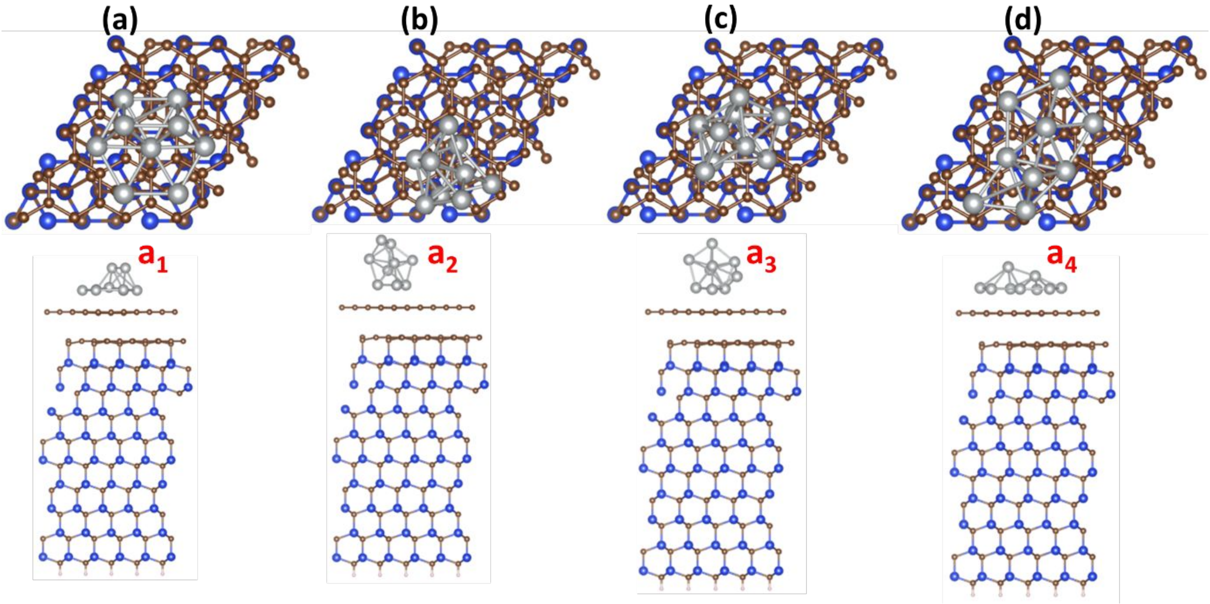

Figure S18. (Top and side views) The optimized geometrical structures of the monolayer epitaxial graphene after adsorption of non-planar Ag_9 clusters (a-d). a_i designates the cluster structure.

Table S1. Energy differences between the lowest energy 2D and 3D ($\Delta E_{2D\rightarrow3D}$) Ag_n clusters on MEG substrate. The negative sign of $\Delta E_{2D\rightarrow3D}$ means that the total energy of 2D Ag_n/MEG structure is more negative than that of 2D Ag_n/MEG structure. The results of charge population analysis are also summarized.

**Cluster size, *n***	**Energy difference, $\Delta E_{2D\rightarrow3D}$, eV**	**Charge transfer (Hirshfeld scheme), e^-**	
		From 2D clusters to MEG	*From 3D clusters to MEG*
2	-0.3155	0.2100	0.0520
3	-0.1646	0.3730	0.3830
4	0.0622	0.5070	0.6510
5	-0.1185	0.4040	0.4490
6	0.1538	0.4380	0.5670
7	-0.1049	0.3410	0.3470
8	0.3142	0.5490	0.5070
9	0.5018	0.4370	0.5150

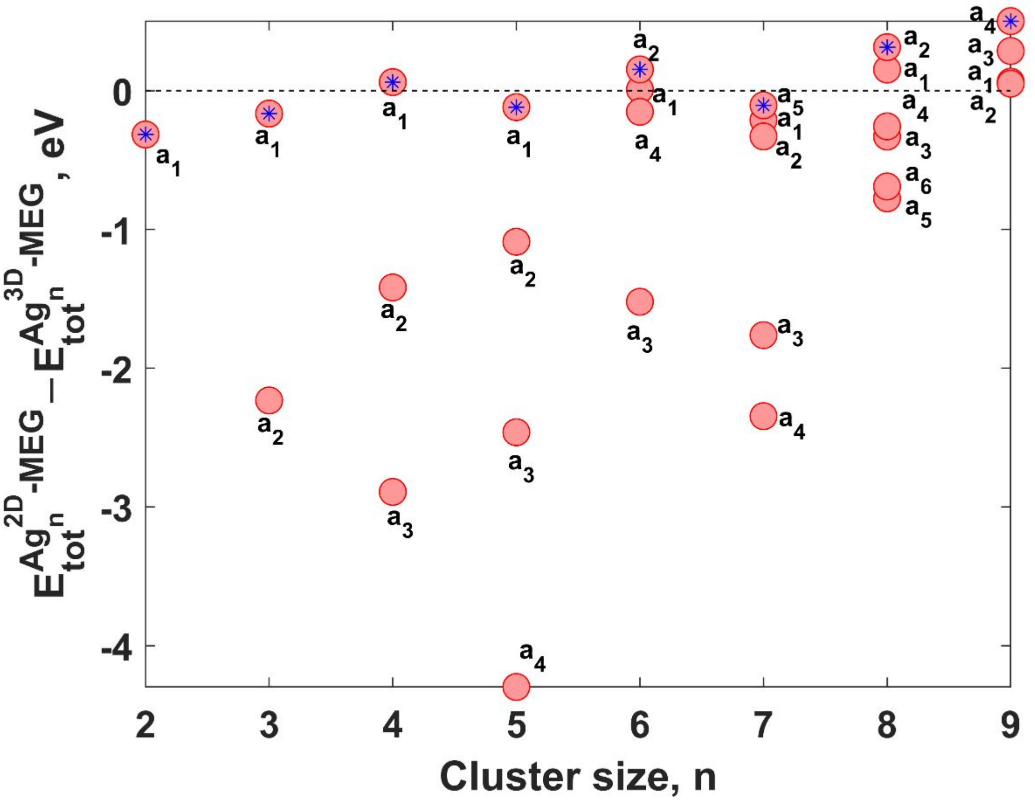

Figure S19. Energy differences between the lowest energy 2D Ag_n clusters and 3D clusters (all configurations) ($\Delta E_{2D\rightarrow3D}$). Blue asterisks designate the lowest-energy 3D Ag_n clusters. a_i for each cluster size *n* corresponds to respective structure demonstrated in Figures S15-S21.

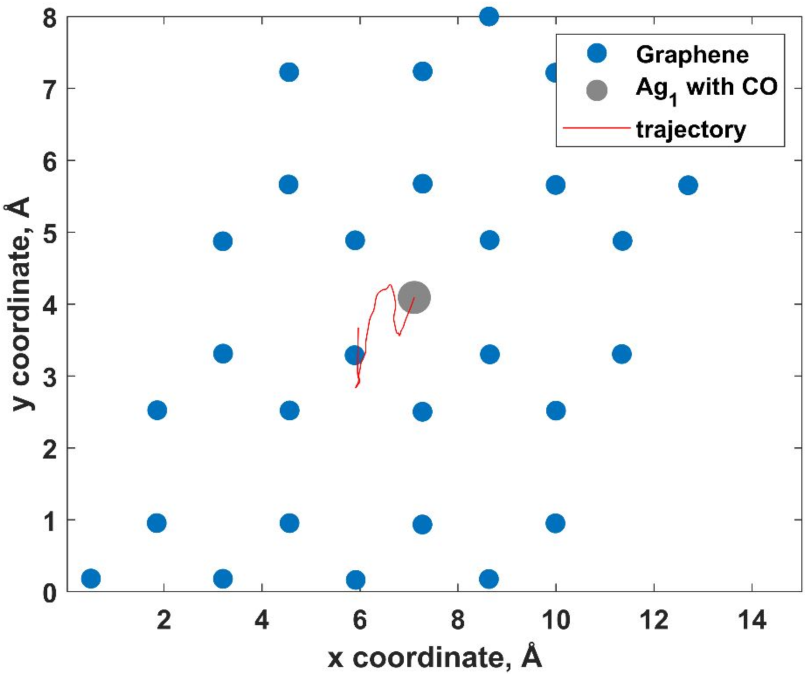

Figure S20. Migration trajectory of Ag_1 monomer onto MEG substrate in the presence of CO predicted by MD simulation at 300 K during 2.1 ps

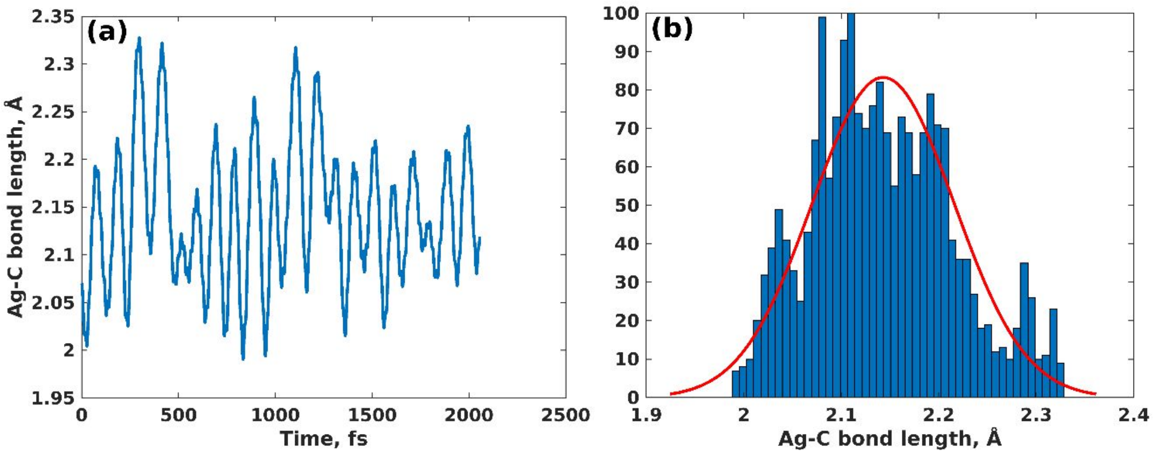

Figure S21. (a) Time evolution of Ag-C bond length for CO-Ag_1-MEG structure. (b) Ag-C bond length distribution histogram fitted with normal (Gaussian) distribution (red solid curve).

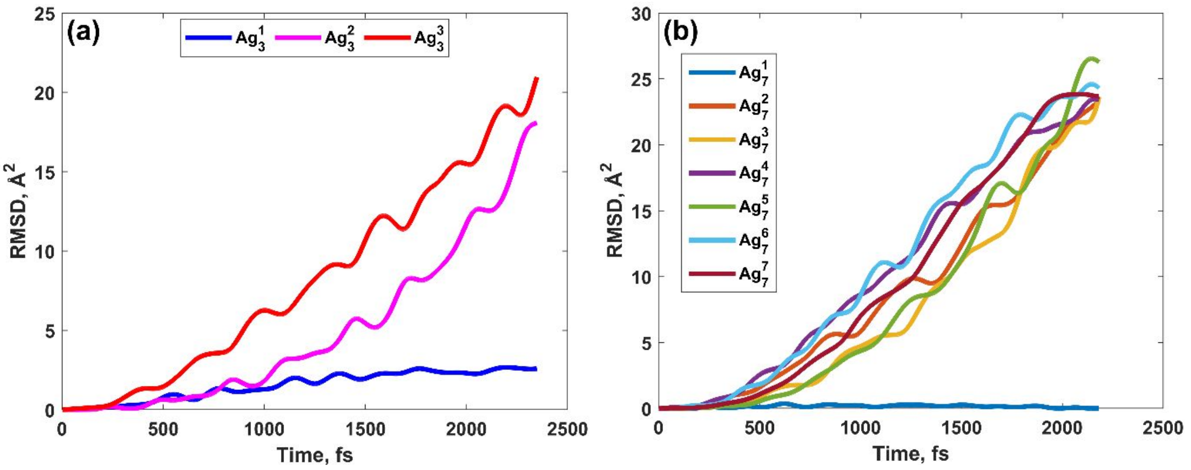

Figure S22. Plots of RMSD vs. time of silver atoms belonging to Ag_3 (a) and Ag_7 (b) clusters on MEG, respectively.

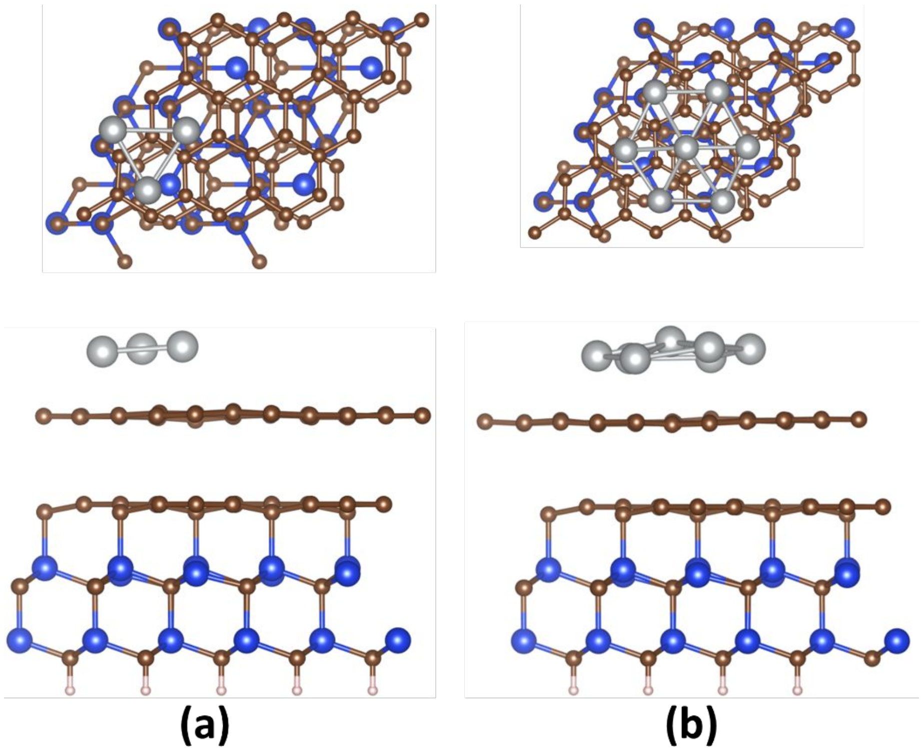

Figure S23. Snapshots from the molecular dynamics simulations at 300 K: (a) Ag_3 and (b) Ag_7 clusters on MEG at t=2.3 ps.

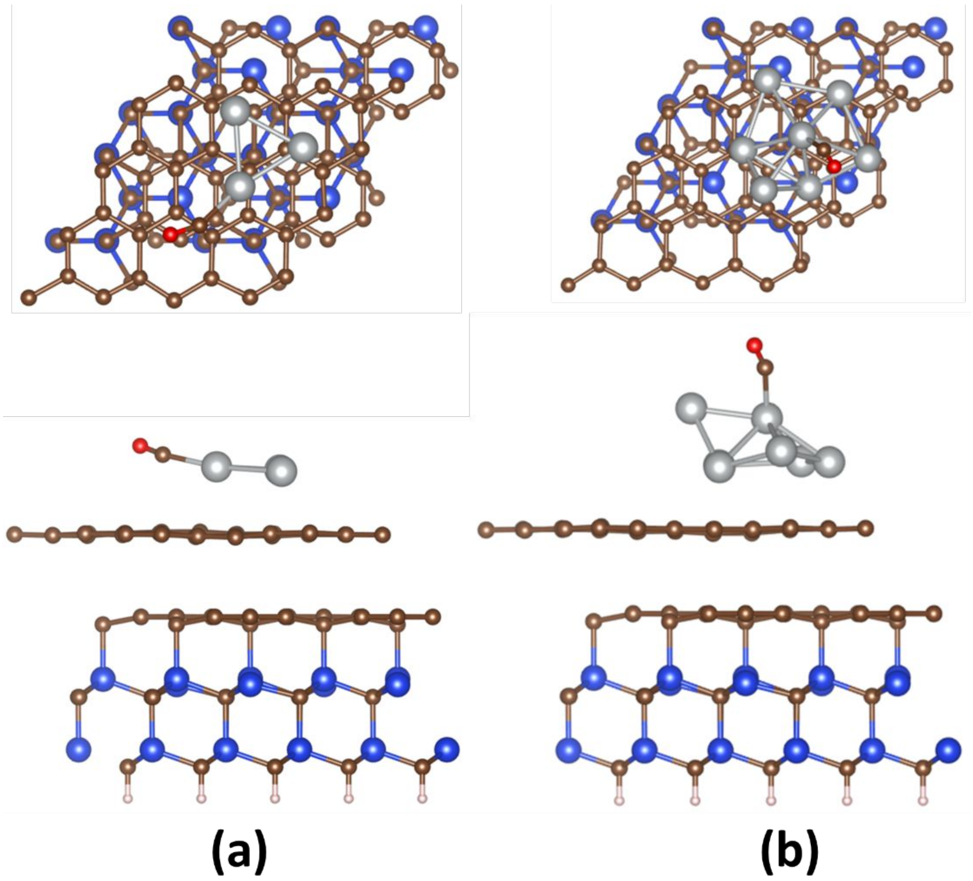

Figure S24. Snapshots from the molecular dynamics simulations at 300 K in the presence of CO molecule: (a) Ag_3 and (b) Ag_7 clusters on MEG at t=2.1 ps.

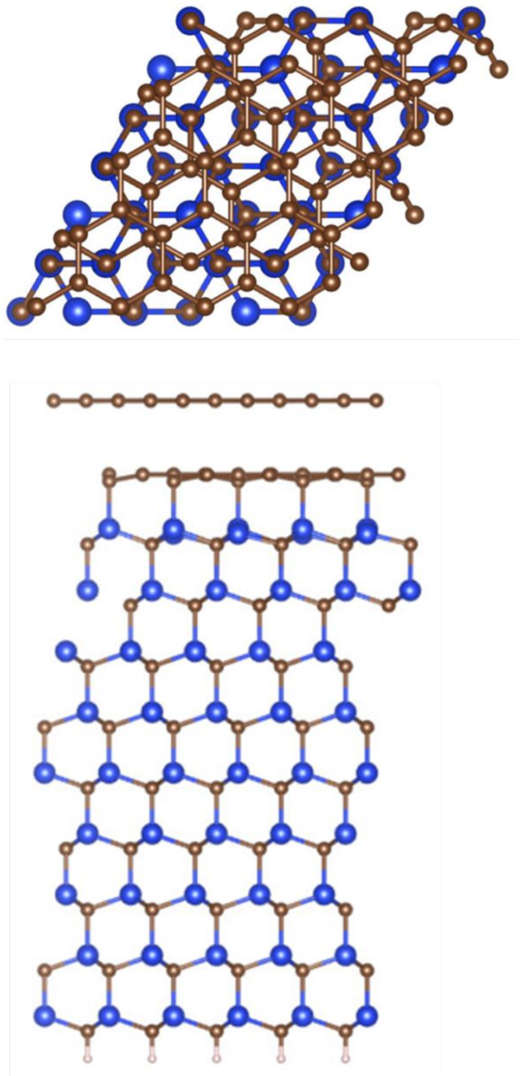

Figure S25. (Top and side views) (Side view) The optimized geometrical structure of 4×4 supercell and monolayer epitaxial graphene. Blue, brown, and whitish balls designate Si, C and H atoms, respectively.

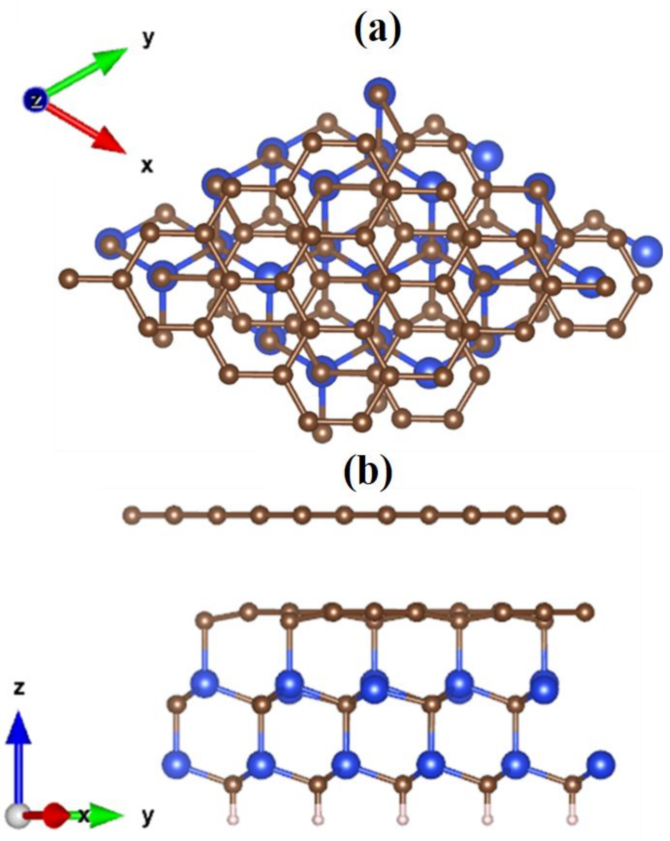

Figure S26. 4×4 epitaxial graphene model with two Si-C bilayers used for MD calculations: (a) top view and (b) side view. Blue, brown, and whitish balls designate Si, C and H atoms, respectively.
